# Vitamin C modulates the metabolic and cytokine profiles, alleviates hepatic endoplasmic reticulum stress, and increases the life span of *Gulo^−/−^* mice

**DOI:** 10.18632/aging.100902

**Published:** 2016-02-20

**Authors:** Lucie Aumailley, Alessandra Warren, Chantal Garand, Marie Julie Dubois, Eric R. Paquet, David G. Le Couteur, André Marette, Victoria C. Cogger, Michel Lebel

**Affiliations:** ^1^ Centre de Recherche du CHU de Québec, Faculty of Medicine, Université Laval, Quebec City Quebec, Canada; ^2^ Centre for Education and Research on Aging and ANZAC Research Institute, University of Sydney and Concord Hospital, New South Wales, Australia; ^3^ Quebec Heart and Lung Institute, Faculty of Medicine, Université Laval, Quebec City, Quebec, Canada; ^4^ Centre de Recherche sur le Cancer de l'Université Laval, Hôpital Hôtel-Dieu de Québec, Quebec City, Quebec, Canada

**Keywords:** vitamin C, gulonolactone oxidase, metabolomic, inflammation, endoplasmic reticulum stress, aging

## Abstract

Suboptimal intake of dietary vitamin C (ascorbate) increases the risk of several chronic diseases but the exact metabolic pathways affected are still unknown. In this study, we examined the metabolic profile of mice lacking the enzyme gulonolactone oxidase (Gulo) required for the biosynthesis of ascorbate. *Gulo^−/−^* mice were supplemented with 0%, 0.01%, and 0.4% ascorbate (w/v) in drinking water and serum was collected for metabolite measurements by targeted mass spectrometry. We also quantified 42 serum cytokines and examined the levels of different stress markers in liver. The metabolic profiles of *Gulo^−/−^* mice treated with ascorbate were different from untreated *Gulo^−/−^* and normal wild type mice. The cytokine profiles of *Gulo^−/−^* mice, in return, overlapped the profile of wild type animals upon 0.01% or 0.4% vitamin C supplementation. The life span of *Gulo^−/−^* mice increased with the amount of ascorbate in drinking water. It also correlated significantly with the ratios of serum arginine/lysine, tyrosine/phenylalanine, and the ratio of specific species of saturated/unsaturated phosphatidylcholines. Finally, levels of hepatic phosphorylated endoplasmic reticulum associated stress markers IRE1α and eIF2α correlated inversely with serum ascorbate and life span suggesting that vitamin C modulates endoplasmic reticulum stress response and longevity in *Gulo^−/−^* mice.

## INTRODUCTION

Maintaining adequate vitamin C (ascorbate) levels in tissues is essential for normal body function and optimal health [1]. Most mammals are capable of synthesizing their own ascorbate and are thus not prone to develop vitamin C deficiency. Humans, however, have a mutation in the gene encoding the enzyme gulono-lactone oxidase (GULO) necessary for the last step of ascorbic acid synthesis [2]. Hence, humans rely entirely on dietary sources to obtain adequate amounts of ascorbate. Ascorbate is a cofactor in several enzymatic reactions, including collagen synthesis that, when dysfunctional, cause scurvy [3]. Although scurvy is now considered a rare disease, epidemiological studies suggest that large subpopulations (between 5% and 30% depending on socioeconomic status, smoking status, and age) can be diagnosed with hypovitaminosis C [3-6]. Even though a hypovitaminosis C condition may not lead to scurvy, it places an individual at higher risk for metabolic abnormalities, cardiovascular diseases or cancer [3, 7-9].

Clinically relevant animal models of ascorbate synthesis deficiency are essential for improving our understanding of the role of vitamin C in the pathogenesis of complex diseases as well as evaluating the therapeutic potential and risks of its supplementation [10]. The progress of gene knockout mice has potentiated these areas of research. In this context, a *Gulo^−/−^* mouse was created by deleting exons 3 and 4 from the *Gulo* gene in a C57BL/6 background [11]. This exonal knockout inactivates the enzyme and ascorbate supplementation is required to maintain viability in these mice [11]. Additional studies on this knockout model indicated elevated oxidative stress and sensorimotor deficits as well as behavioral and monoamine changes following severe ascorbate deficiency [12-15]. Furthermore, it has recently been observed that ascorbate prevents stress-induced damage on the heart through the reduction of reactive oxygen species production in *Gulo^−/−^* mice depleted of ascorbate [16]. Metabolic profiling of ascorbate deficiency in *Gulo^−/−^* mice using proton NMR spectroscopy revealed changes in carnitine and glutathione synthesis as well as changes in glycerophospholipid metabolism [17]. In this study, we have extended this research by measuring amino acids, biogenic acids, acylcarnitines, lyso-phosphatidylcholines, glycerophosphatidylcholines, sphingomyelins, and prostaglandins in the serum of *Gulo^−/−^* mice treated with different concentrations of ascorbate in drinking water by targeted mass spectrometry analysis. We also examined the levels of several inflammatory cytokines, metabolic hormones, and markers of cardiovascular diseases in these animals and determined the molecules that significantly correlated with life span. We found that the median life span of *Gulo^−/−^* mice increased with the amount of ascorbate in drinking water. Although the average size of mitochondria in the liver of ascorbate depleted *Gulo^−/−^* mice was increased, overall mitochondrial alterations did not correlate with median life span in our mouse cohorts. In contrast, the amount of phosphorylated endoplasmic reticulum associated stress markers inositol-requiring kinase 1α (IRE1α) and the eukaryotic translation initiation factor 2α (eIF2α) in the hepatic tissues of *Gulo^−/−^* mice treated with different concentrations of ascorbate significantly correlated inversely with serum ascorbate levels and median life span.

## RESULTS

### Impact of vitamin C (ascorbate) on life span and body weight of *Gulo^−/−^* mice

**Figure 1:**
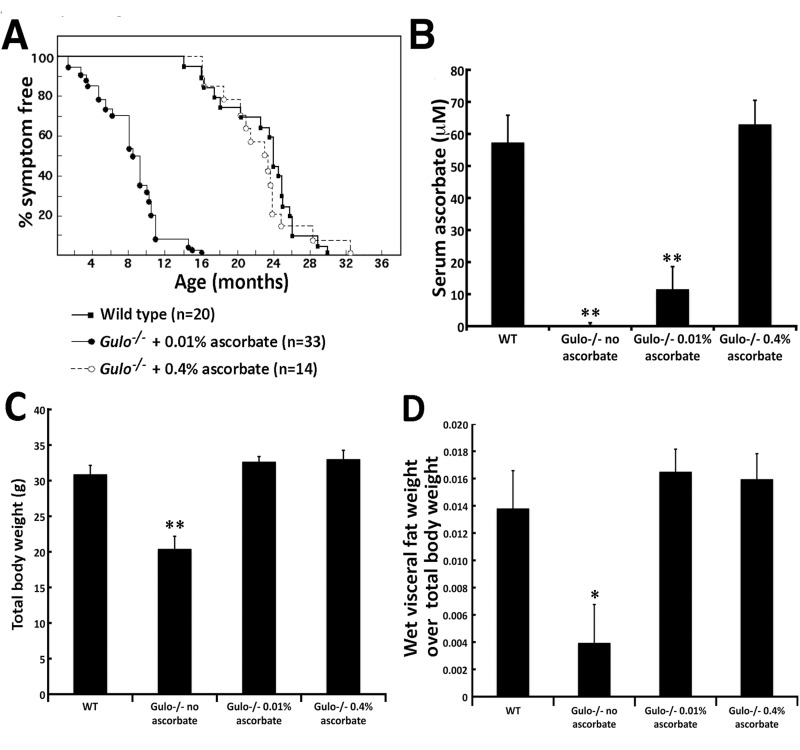
Impact of vitamin C (ascorbate) on the life span and the body weight of *Gulo^−/−^* mice **(A)** Percentage of disease-free animals with age. The number of animals in each group is indicated. *Gulo^−/−^* mice were treated with the indicated concentration of ascorbate in drinking water from weaning until they had to be euthanized due to illness. Wild type mice were not treated with ascorbate. **(B)** Serum ascorbate levels in each indicated cohort (n=5 males at four months of age). (Tukey post ANOVA test **P < 0.01 compared to wild type and 0.4% treated *Gulo^−/−^* mice). **(C)** Histogram showing the mean total body weight of each mouse cohort (n=6 males) at four month of age. (Tukey post ANOVA test **P < 0.01 compared to all other groups). **(D)** Histogram showing a significant visceral fat weight loss in ascorbate deficient *Gulo^−/−^* mice (n=6 males) at four month of age. (Tukey post ANOVA test *P < 0.05 compared to all other groups). (**B-D**) Bars in all histograms represent SEM. One cohort of *Gulo^−/−^* mice was treated with 0.01% of ascorbate (w/v) in drinking water from weaning to four months of age. A second cohort of *Gulo^−/−^* mice was treated with 0.4% of ascorbate from weaning to four months of age. In a third cohort of *Gulo^−/−^* mice, ascorbate was omitted from the drinking water at the age of three months for four weeks. Wild type mice were not treated with ascorbate.

We first investigated the effect of ascorbate supplementation on the median life span of *Gulo^−/−^* mice. Generally, four to six weeks after removal of ascorbate in drinking water, *Gulo^−/−^* mice lost more than 20% of their total body weight, looked moribund, and had to be euthanized. In contrast, increasing ascorbate concentration in drinking water improved the survival of these mutant mice in a dose dependent manner (Figure 1A). *Gulo^−/−^* mice supplemented with 0.005% of ascorbate (w/v) at weaning rapidly became sick and moribund within four weeks of treatment (data not shown). Therefore this concentration of ascorbate was not used for further analysis. The median life span of *Gulo^−/−^* mice treated with 0.01% ascorbate was 8 ½ months and the maximum life span was 16 months. All of these animals lost more than 20% of their total body weight, became moribund at some point during this 16 months period and had to be euthanized. Supplementation of ascorbate up to 0.4% (w/v) significantly increased the median life span to 23 months, which was close to the median life span of wild type mice (23.8 months). The maximum life span *Gulo^−/−^* mice treated with 0.4% ascorbate was ∼32 months compared to ∼30 months for untreated wild type animals. The illnesses associated with old *Gulo^−/−^* mice treated with 0.4% vitamin C included either hepatocarcinomas or myeloid leukemias. Several mice were also found dead in the cages in the morning with no sign of distress in the previous days (undetermined cause of death due to tissue autolysis). Overall, these results indicated that the life span of *Gulo^−/−^* mice increased with ascorbate concentration in drinking water. The amount of ascorbate required to achieve a wild type normal life span (log rank test *P* > 0.05) was reached with 0.4% ascorbate in drinking water (Figure 1A). As we have previously found that 0.4% ascorbate did not increase the life span of wild type animals [18], we did not pursue analyses with higher concentrations of ascorbate in the drinking water of *Gulo^−/−^* mice.

Next we measured the amount of ascorbate in the serum of mice from each treatment group (n=5). As indicated in Figure 1B, minimal ascorbate could be detected in the serum of ascorbate-depleted *Gulo^−/−^* mice (0% of ascorbate in drinking water for one month). There was a 100-fold significant increase in serum ascorbate in *Gulo^−/−^* mice treated with 0.01% ascorbate compared to *Gulo^−/−^* mice that had been depleted of ascorbate for one month (*P* < 0.01). Furthermore, there was a 5.4-fold increase when *Gulo^−/−^* mice were supplemented with 0.4% ascorbate compared to 0.01% ascorbate treated mice (*P* < 0.01). *Gulo^−/−^* mice treated with 0.4% ascorbate had similar levels of serum ascorbate compared to wild type untreated animals (Figure 1B).

There was a significant effect of ascorbate supplementation on body weight in the *Gulo* ^−/−^ animals. As indicated in Figure 1C, *Gulo^−/−^* mice without ascorbate treatment were significantly leaner than *Gulo^−/−^* mice treated with 0.01% or 0.4% ascorbate. The mean total body weight of *Gulo^−/−^* mice treated with 0.01% or 0.4% ascorbate at four months of age were not significantly different from age-matched wild type animals. Analysis of the four-month old animals revealed that *Gulo^−/−^* mice depleted of ascorbate for one month had significantly less visceral fat in proportion to total body weight compared to all *Gulo^−/−^* mice treated with ascorbate (Figure 1D). The weight of visceral fat in ascorbate treated *Gulo^−/−^* mice (0.01% or 0.4% in drinking water) was not significantly different from untreated wild type animals (Figure 1D).

The spleen, kidneys, heart, and liver were also weighed (Supplementary Figure S1). The spleen tended to be larger in all the *Gulo^−/−^* mice compared to age-matched four-month old wild type animals. However, only the *Gulo^−/−^* mice treated with 0.01% ascorbate showed a significant difference compared to wild type animals (Supplementary Figure S1A). The weight of the kidneys in *Gulo^−/−^* mice depleted of ascorbate was increased (∼1.2-fold) compared to age-matched wild type but was significantly decreased in *Gulo^−/−^* mutant mice treated with 0.01% or 0.4% ascorbate (Supplementary Figure S1B). The weights of the cardiac and hepatic tissues were not significantly different between cohorts (Supplementary Figure S1C and D).

Finally, *Gulo^−/−^* mice depleted of ascorbate for one month ate and drank less than wild type (based on student *t*-test: P < 0.05; supplementary Figure S1E and F).

### Effect of ascorbate on the metabolic profile of *Gulo^−/−^* mice

**Figure 2:**
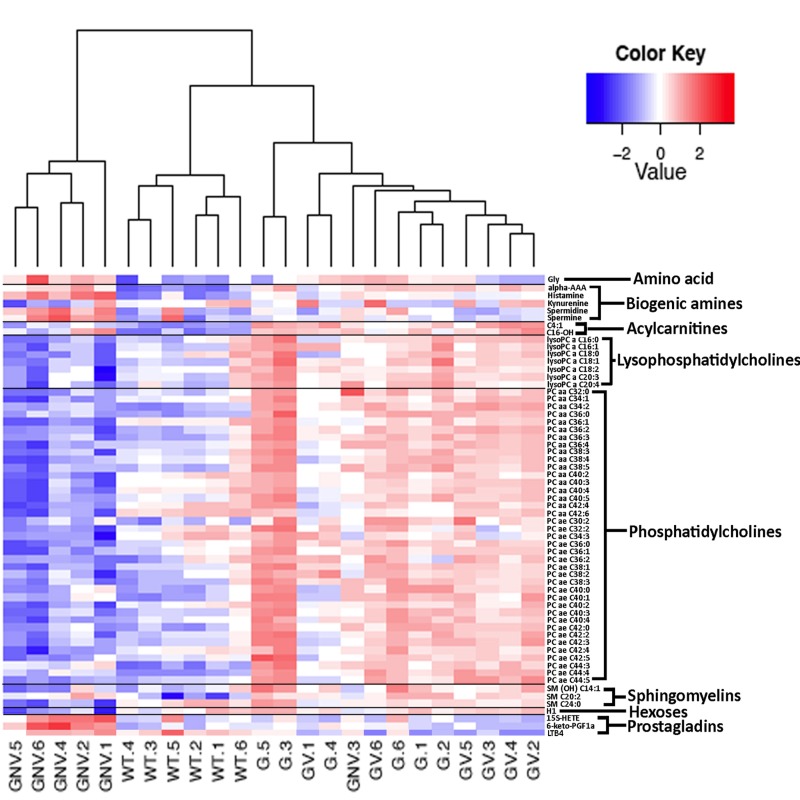
Heatmap depicting the Z-score of the log base ten in serum metabolites concentration (rows) between individual (columns) wild type and *Gulo^−/−^* mice treated with different amounts of ascorbate Columns are reordered by hierarchical clustering using the genotype and ascorbate treatments to label the three leaves. Metabolites are grouped according to chemical classification. Wild type animals are labeled WT.1 to WT.6. *Gulo^−/−^* mice treated with 0.4% ascorbate in drinking water are labeled GV.1 to GV.6. *Gulo^−/−^* mice treated with 0.01% ascorbate are labeled G.1 to G.6. *Gulo^−/−^* mice treated with 0% ascorbate in drinking water from the age of three to four months are labeled GNV.1 to GNV.6. The Euclidean distance and complete agglomerative methods were used for clustering.

**Figure 3:**
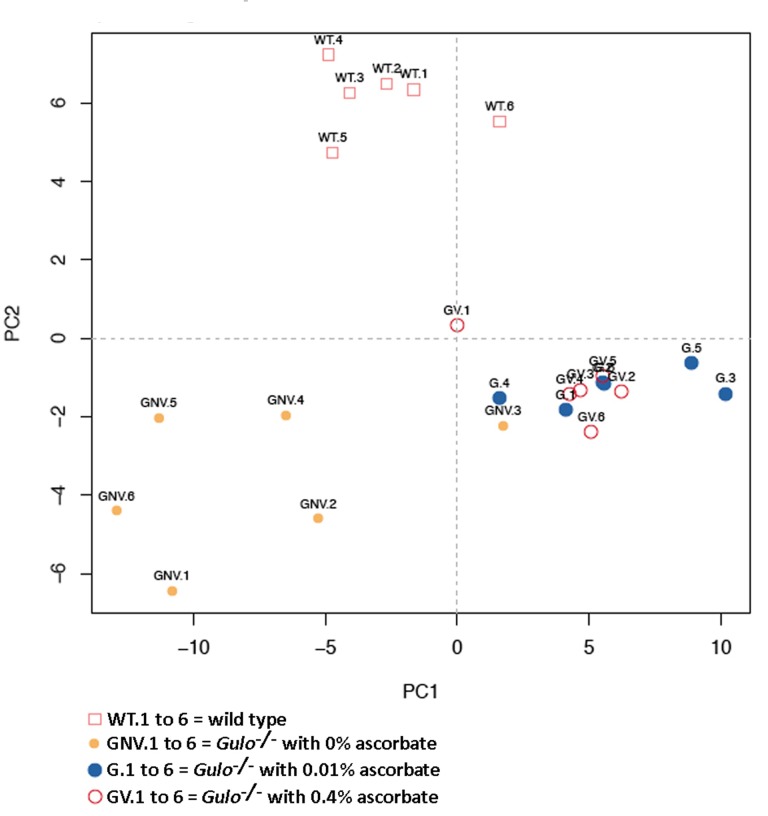
Principal component analysis (PCA) graph demonstrating the differentiation effect of ascorbate on the metabolomic profiles of wild type and *Gulo^−/−^* mice treated with different amounts of ascorbate X axis: Principal component 1, Y axis: Principal component 2. Wild type animals are labeled WT.1 to WT.6. *Gulo^−/−^* mice treated with 0.4% ascorbate in drinking water are labeled GV.1 to GV.6. *Gulo^−/−^* mice treated with 0.01% ascorbate are labeled G.1 to G.6. *Gulo^−/−^* mice treated with 0% ascorbate in drinking water from the age of three to four months are labeled GNV.1 to GNV.6.

We next measured 203 metabolites employing a targeted mass spectrometry approach in the serum of *Gulo^−/−^* mice treated with different amounts of ascorbate at four months of age. (Full biochemical names are provided in Supplementary Table S1). Untreated age-matched wild type animals were used as the reference control. Serum metabolite concentrations in six animals of each group are shown in the supplementary Table S2. To identify the metabolites significantly altered in either wild type, *Gulo^−/−^* mice treated with 0%, 0.01%, or 0.4% ascorbate, a nonparametric Kruskal-Wallis test was applied to the data. A summary of the metabolites significantly altered (*P* < 0.01) in at least one of the groups is given in a form of a heatmap in Figure 2. Changes were indicated as a Z-score value for each metabolite in the serum of each animal. The heatmap revealed 61 metabolites that significantly differed between groups (Figure 2). These included 39 phosphatidylcholines, seven lysophosphatidylcholines, two acylcarnitines, five biogenic amines, three prosta-glandins, three sphingomyelins, hexoses, and the amino acid glycine. The dendrogram on top of the heatmap indicated that the *Gulo^−/−^* mice depleted of ascorbate clustered together except for one mouse (mouse identified as GNV.3). The wild type animals also formed a cluster. *Gulo^−/−^* mice supplemented with either 0.01% or 0.4% ascorbate in their drinking water formed another cluster. We further explored the metabolic profile of each group by employing principal component analysis (PCA in Figure 3). The PCA approach showed a good clustering of *Gulo^−/−^* mice supplemented with either 0.01% or 0.4% ascorbate in the lower right quadrant of the graph. Five *Gulo^−/−^* mice depleted of ascorbate were found in the lower left quadrant (and one animal in the lower right quadrant). All wild type animals were found in the upper quadrants of the graph and did not overlap with any mouse of the *Gulo^−/−^* genotype. The PCA analysis indicated that although there was a larger variability within the ascorbate depleted *Gulo^−/−^* mouse group, they were localized at great distance from the wild type mice in the graph (Figure 3). The clustering of *Gulo^−/−^* mice supplemented with either 0.01% or 0.4% ascorbate suggested similar metabolic profiles at four months of age. Such profiles were different from both wild type and ascorbate depleted *Gulo^−/−^* mice. Finally, even though *Gulo^−/−^* mice supplemented with 0.4% ascorbate had a life span and serum ascorbate levels similar to wild type animals (Figure 1A and B), their metabolic profile did not significantly overlap with any wild type individual (Figure 3).

### Effect of ascorbate on specific metabolites in *Gulo^−/−^* mice

The levels of phosphatidylcholines, lysophosphatidyl-cholines, sphingomyelins, and hexoses were generally lower in the ascorbate deficient *Gulo^−/−^* mice compared to wild type mice. Supplementation with ascorbate increased these levels equal to or above the wild type levels (Figure 2 and supplementary Table S2). The 15S-HETE (15(S)-hydroxy-5Z,8Z,11Z,13E-eicosatetraenoic acid) and 6-keto-PGF1a (6-keto prostaglandin F1α) were significantly up regulated in the serum of ascorbate deficient *Gulo^−/−^* mice compared to wild type animals (Figure 2 and supplementary Table S2). Supplementation of drinking water with 0.4% ascorbate decreased both serum 15S-HETE and 6-keto-PGF1a to wild type levels in *Gulo^−/−^* mice (Figure 2 and supplementary Table S2). LTB4 (leukotriene B4) was lower in ascorbate deficient *Gulo^−/−^* mice compared to wild type animals. Supplementation of ascorbate in drinking water decreased serum LTB4 even further in *Gulo^−/−^* mice (Figure 2 and supplementary Table S2). Four biogenic amines (spermine, spermidine, histamine, and alpha-aminoadipic acid) were significantly increased in ascorbate deficient *Gulo^−/−^* mice compared to wild type animals (Figure 2 and supplementary Table S2). Histamine, spermine and its precursor spermidine were decreased to normal wild type levels when *Gulo^−/−^* mice were treated with 0.4% ascorbate (Figure 2 and supplementary Table S2). In contrast, the α-aminoadipic acid (alpha-AAA) remained elevated in *Gulo^−/−^* mice compared to wild type animals in both the absence and presence of ascorbate in drinking water (Figure 2 and supplementary Table S2). Finally, the amino acid glycine was significantly increased in ascorbate deficient *Gulo^−/−^* mice compared to wild type animals (Figure 2 and supplementary Table S2). Supplementation of drinking water with 0.4% ascorbate in *Gulo^−/−^* mice decreased serum glycine to wild type levels.

### Effect of ascorbate on inflammatory cytokines and metabolic hormones in *Gulo^−/−^* mice

**Figure 4:**
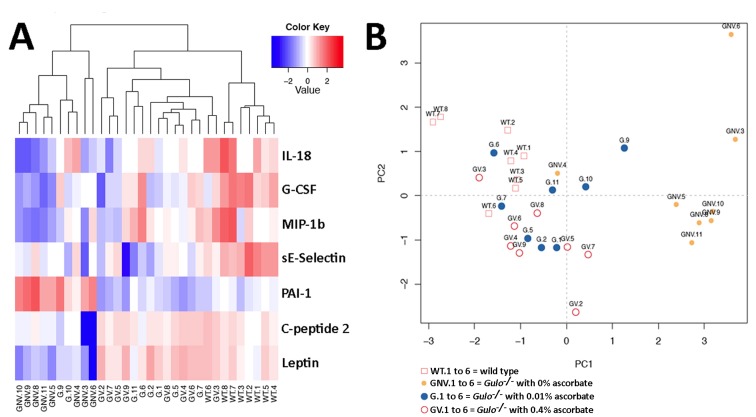
Impact of ascorbate on the cytokinome of *Gulo^−/−^* mice **(A)** Heatmap depicting the Z-score of log base ten in serum cytokine concentrations (rows) between individual (columns) wild type and *Gulo^−/−^* mice treated with different amounts of ascorbate. Columns are reordered by hierarchical clustering using the genotype and ascorbate treatments to label the three leaves. **(B)** Principal component analysis (PCA) graph demonstrating the differentiation effect of ascorbate on the cytokinome profiles of wild type and *Gulo^−/−^* mice treated with different amounts of ascorbate. Wild type animals are labeled WT.1 to WT.6. *Gulo^−/−^* mice treated with 0.4% ascorbate in drinking water are labeled GV.1 to GV.6. *Gulo^−/−^* mice treated with 0.01% ascorbate are labeled G.1 to G.6. *Gulo^−/−^* mice treated with 0% ascorbate in drinking water from the age of three to four months are labeled GNV.1 to GNV.6. The Euclidean distance and complete agglomerative methods were used for clustering.

As metabolite disturbances can lead to an inflammatory response or changes in cardiovascular risk factors, we measured the levels of 42 cytokines (including hormones) in the serum of our different mouse cohorts. Serum cytokine concentrations in eight males of each group are shown in the supplementary Table S3. To identify the cytokines significantly altered in either wild type, *Gulo^−/−^* mice treated with 0%, 0.01%, or 0.4% ascorbate, the nonparametric Kruskal-Wallis test was applied to the data. A summary of the metabolites significantly altered (*P* < 0.01) in at least one of the groups is given in a form of a heatmap in Figure 4A. Changes were indicated as a Z-score value for each metabolite in the serum of each animal. The dendrogram on top of the heatmap indicated that the *Gulo^−/−^* mice depleted of ascorbate tended to cluster together. The wild type animals also tended to form a cluster. We further explored the cytokine profile of each group by employing principal component analysis (PCA in Figure 4B). The PCA approach showed that wild type mice were mainly clustered in the upper left quadrant of the graph. *Gulo^−/−^* mice supplemented with either 0.01% or 0.4% ascorbate were distributed in all four quadrants but their localization overlapped with the wild type animals. Ascorbate deficient *Gulo^−/−^* mice were localized on the right side of the graph (except for mouse GNV.4). To summarize the PCA data, the serum cytokine profile of the ascorbate deficient *Gulo^−/−^* mice was quite different from ascorbate treated *Gulo^−/−^* and wild type mice.

The heatmap revealed seven cytokines that significantly differed between groups (Figure 4A). These included three pro-inflammatory cytokines (interleukin-18, granulocyte-colony stimulating factor, and the macrophage inflammatory protein 1 beta), two metabolic hormones (leptin and C-peptide 2), and two cardiovascular risk factors (secreted E-selectin and the plasminogen activator inhibitor-1). The most marked findings were a general decrease in the inflammatory factors and sE-selectin in ascorbate deficient *Gulo^−/−^* mice compared to wild type animals (summarized as graphs in the supplementary Figure S2). Ascorbate treatment increased the levels of these cytokines but to a level still below wild type animals. The hormones leptin and C-peptide 2 were significantly decreased in ascorbate depleted *Gulo^−/−^* mice. Supplementation of ascorbate reversed the phenotype. Finally, the plasminogen activator inhibitor-1 (PAI-1) was significantly increased in ascorbate depleted *Gulo^−/−^* mice compared to wild type animals (supplementary Figure S2). Supplementation of ascorbate in drinking water also reversed this phenotype in *Gulo^−/−^* mice.

### Correlation between cytokines and metabolites in the different *Gulo^−/−^* cohorts

**Figure 5:**
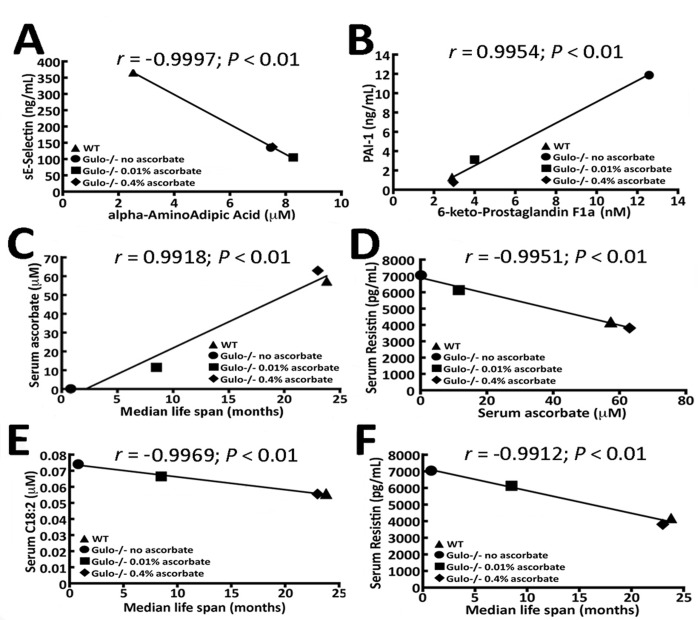
Correlation of median life span and serum ascorbate levels in *Gulo^−/−^* mice with different metabolites and cytokines **(A)** Correlation between alpha-AAA and sE-selectin with a *r* > 0.99 and a *P*-value < 0.01 in our mouse cohorts. **(B)** Correlation between 6-keto-PGF1a and PAI-1 with a *r* > 0.99 and a *P*-value < 0.01 in our mouse cohorts. **(C)** Graphs showing the correlation between the median life span of mice and serum ascorbate level. **(D)** Correlation between serum ascorbate and resistin. **(E)** Correlation between serum acylcarnitine C18:2 and median life span. **(F)** Correlation between resistin and median life span. The Pearson's correlation *r* values and *P*-values are indicated on each graph.

We determined whether there were any strong correlations (*r* > 0.99 and *P* < 0.01) between the cytokines and any of the metabolites that were altered in the *Gulo^−/−^* cohorts. Only two cytokines passed such stringent criteria for these correlations. The serum levels of alpha-aminoadipic acid (alpha-AAA) showed an inverse correlation with serum sE-Selectin (Figure 5A), while the levels of serum 6-keto-PGF1a was positively correlated with serum plasminogen activator inhibitor-1 (PAI-1) (Figure 5B).

### Correlation between ascorbate and the median life span of *Gulo^−/−^* mice with metabolites or cytokines

We then determined which metabolites and cytokines were correlated with the median life span of our *Gulo^−/−^* cohorts. *Gulo^−/−^* mice without ascorbate in the drinking water lived approximately one month as indicated earlier. The median life span of wild type and *Gulo^−/−^* mice treated with either 0.01% or 0.4% ascorbate was obtained from the graph in Figure 1A. As such, the median life span of *Gulo^−/−^* mice correlated positively with serum ascorbate levels (*r* = 0.9918; *P* < 0.01 in Figure 5C). The molecule that showed strongest correlation with serum ascorbate level was serum resistin (Figure 5D). Serum resistin levels were inversely correlated with serum ascorbate levels.

The molecules that were correlated to median life span included serum levels of octadecadienoylcarnitine (C18:2) and resistin (Figure 5E and F). C18:2 and resistin exhibited an inverse correlation with the median life span in our mouse cohorts (with a *r* of at least −0.99 and a *P* < 0.01). Other metabolites and cytokines measured in the serum of our mouse cohorts did not correlate to ascorbate levels with a Pearson's correlation *r* value > 0.99. Although we obtained high Pearson's correlation *r* values between the median life span and the mean levels of serum resistin and C18:2, these molecules exhibited large standard deviations from the means within each group of mice such that differences between groups were not statistically significant (Kruskal-Wallis *P*-values > 0.01). In fact, no individual metabolite (with a Kruskal-Wallis *P*-values > 0.01) correlated with life span and/or ascorbate (even with a *r* value > 0.95 and *P*-value < 0.05). Next we examined specific ratios of metabolites in our cohorts of mice that could correlate with ascorbate levels or median life span with a *r* value > 0.95 and *P*-value < 0.05. Specific metabolite ratios in the serum of mice can provide insight into biological processes relevant to the activity and metabolism of ascorbate.

**Figure 6:**
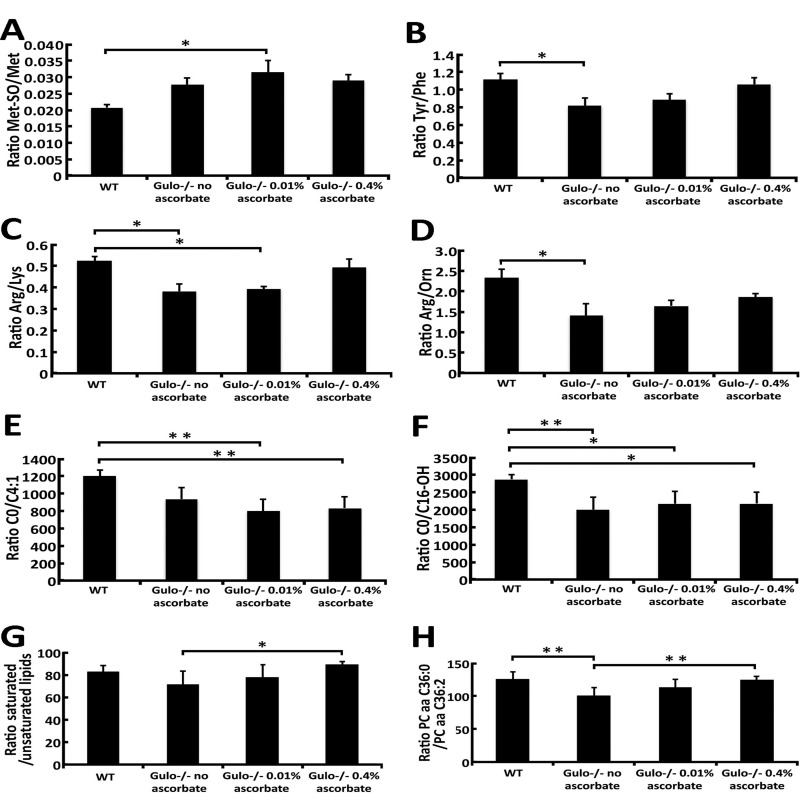
Ratios of metabolites demonstrating significant differences between *Gulo^−/−^* mice treated with different concentrations of ascorbate **(A)** Graph showing the Met-SO/Met ratio in the different groups of mice. Tukey post ANOVA test *P-value < 0.05. **(B)** Graph showing the Tyr/Phe ratio in the different groups of mice. Tukey post ANOVA test *P-value < 0.05. **(C)** Graph showing the Arg/Lys ratio in the different groups of mice. Tukey post ANOVA test *P-value < 0.05. **(D)** Graph showing the Arg/Orn ratio in the different groups of mice. Tukey post ANOVA test *P-value < 0.05. **(E)** Graph showing the C0/C4:1 ratio in the different groups of mice. Tukey post ANOVA test **P-value < 0.01. **(F)** Graph showing the C0/C16-OH ratio in the different groups of mice. Tukey post ANOVA test *P*-values are shown (*P < 0.05 and **P < 0.01). **(G)** Graph showing the ratio of saturated/unsaturated phosphatidylcholine species from the Figure 2. Tukey post ANOVA test *P-value < 0.05. **(H)** Graph showing the PC aa C36:0/PC aa C36:2 ratio in the different groups of mice. Tukey post ANOVA test **P-value < 0.01.

Figure 6 provides metabolite ratios that showed significant difference between groups of mice (with ANOVA *P*-values < 0.05). Methionine-sulfoxide (Met-SO) can be generated via a two-electron-dependent mechanism and the ratio of Met-SO/Met in the serum can be regarded as a marker of oxidative stress [19]. As indicated in Figure 6A, *Gulo^−/−^* mice treated with 0.01% ascorbate showed a significant increase in this ratio compared to wild type animals. Ascorbate is also known to affect the hydroxylation of phenylalanine into tyrosine [20]. Figure 6B indicates that the ratio of Tyr/Phe is significantly reduced in ascorbate depleted *Gulo^−/−^* mice compared to wild type animals. Arginine is the only source for nitric oxide production, a molecule with important vasoprotective and anti-atherosclerotic properties [21]. Because lysine uses the same transport system as arginine for intracellular transport, intracellular arginine availability can potentially be affected by lysine [21]. As indicated in Figure 6C, the Arg/Lys ratio is significantly diminished in vitamin C depleted and in *Gulo^−/−^* mice treated with 0.01% ascorbate. Hepatic arginase will also affect the levels of arginine by catabolizing it into ornithine [21]. As indicated in Figure 6D, the Arg/Orn ratio is significantly decreased in ascorbate depleted *Gulo^−/−^* mice compared to wild type animals. Acylcarnitines are important molecules for mitochondrial function and lipid metabolism. Two acylcarnitine species were significantly altered in our mouse cohorts, namely C4:1 and C16-OH (based on Figure 2). Although levels of C4:1 and C16-OH did not correlate significantly with ascorbate levels or median life span, we examined the ratio of carnitine over these two acylcarnitine species. As indicated in Figure 6E and F, the C0/C4:1 and C0/C16-OH ratios were reduced in all *Gulo^−/−^* mice. Finally, we examined the ratio of saturated/unsaturated phosphatidylcholine species. We pooled the serum concentrations of unsaturated phosphatidylcholines together and saturated phosphatidylcholines together to calculate the ratio. (For example, the concentrations of PC aa C32:0, PC aa C36:0, PC ae C36:0, PC ae C40:0, and PC ae C42:0 that are significantly changed in our mouse cohorts based on Figure 2 were added together to obtain the numerator of the ratio). As indicated in the Figure 6G, the ratio of saturated/unsaturated phosphati-dylcholines was significantly increased in *Gulo^−/−^* mice treated with 0.4% ascorbate compared to ascorbate depleted *Gulo^−/−^* mice. Interestingly, among the different species of lipids altered in our mouse cohorts (Figure 2), the specific ratio PC aa C36:0/PC aa C36:2 showed a significant decrease in ascorbate depleted *Gulo^−/−^* mice that was completely reversed by 0.4% ascorbate (Figure 6H). We calculated the Pearson's correlation *r* values to identify the metabolite ratios that correlated significantly with serum ascorbate levels and/or the median life span of our mice. As indicated in Table 1, the ratios of Arg/Lys, Tyr/Phe, saturated/unsaturated lipids, and PC aa C36:0/PC aa C36:2 correlated significantly with median life span. Serum ascorbate levels correlated significantly with the Arg/Lys, Tyr/Phe, and PC aa C36:0/PC aa C36:2 ratios.

**Table 1:** Pearson's correlation *r* values between serum ascorbate level, median life span and meta-bolite ratios showing significant difference (*P* < 0.05) between *Gulo^−/−^* mice treated with different concentrations of ascorbate

	Arg/Lys	Tyr/Phe	saturated/unsaturated^a^	PC aa C36:0/PC aa C36:2
Ascorbate	0.9656	0.9737	not significant	0.9516
Median life span	0.9524	0.9768	0.9527	0.9828

a Ratio of pooled saturated/pooled unsaturated phosphatidylcholine concentrations from Figure 2.

### No correlation between the median life span of *Gulo^−/−^* mice and body weight

Since body mass and visceral fat may have an effect on life span, we determined whether they were significantly correlated in our mouse cohorts. As expected, visceral fat wet weight correlated significantly with total body weight (*r* = 0.9972; *P* < 0.01) in these mice. The median life span of *Gulo^−/−^* mice, however, did not correlate with the mean total body weight of mice or the visceral wet weight/body weight ratio (Table 2). The median life span did not correlate with the weight of the other organs. Similarly, serum ascorbate levels did not significantly correlate with total body weight, visceral fat weight or the weight of any organ analyzed (Table 2).

**Table 2:** Pearson's correlation *r* values between median life span and organ wet weight

	Ascorbate	Total BW	Visceral fat^a^	Spleen^b^	Kidney^c^	Heart^d^
Median life span	0.9918*	0.7335	0.6808	−0.5668	−0.6745	−0.6828
Ascorbate	-	0.6515	0.5925	−0.5417	−0.6204	−0.6674
Total body weight (BW)	-	-	0.9972*	−0.0262	−0.9725^†^	−0.9019

a Ratio of visceral fat wet weight over total body weight.

b Ratio of spleen wet weight over total body weight.

c Ratio of kidney wet weight over total body weight.

d Ratio of heart wet weight over total body weight.

* Pearson's correlation *r* value with a *P*-value < 0.01.

† Pearson's correlation *r* value with a *P*-value < 0.05.

Visceral fat wet weight (and thus total body weight) significantly correlated with the levels of several serum phosphatidylcholine lipid species and serum hexoses (supplementary Table S4). Such phosphatidylcholine lipid species and hexoses, however, did not correlate significantly with the median life span of our mice (*P*-values > 0.05).

### Correlation between hepatic mitochondrial morphology and metabolites or cytokines in *Gulo^−/−^* mice

The liver plays a pivotal role in nutrient, hormone, lipid, and metabolic waste product processing, thereby maintaining body homeostasis [22]. It is also the normal site of ascorbate synthesis in mice [2]. Since ascorbate treated *Gulo^−/−^* mice exhibited alterations in several lipid species, we next measured several cellular morpholo-gical parameters in the hepatic tissue. We first examined sinusoidal endothelial fenestration in liver of mice. Liver endothelial defenestration is a recognized age-related change [23]. Scanning electron microscopy revealed no significant difference in the number of sinusoidal endothelial fenestration between our cohorts of mice (supplementary Figure S3A). In addition, fenestration diameter was not significantly different between cohorts (supplementary Figure S3B and C).

**Figure 7:**
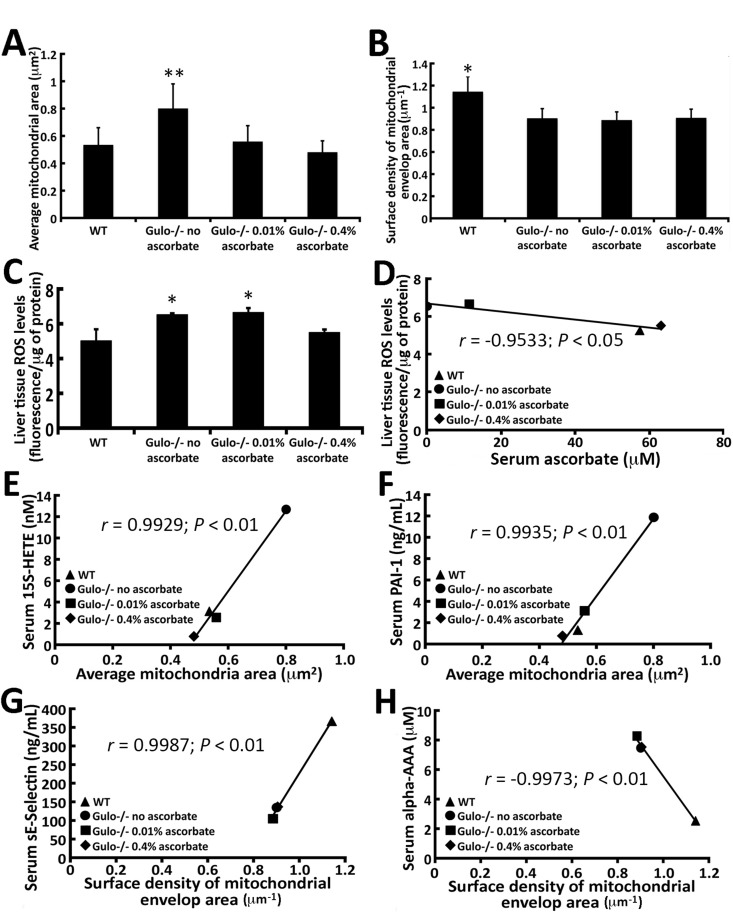
Correlation of mitochondrial morphology in the liver of *Gulo^−/−^* mice with different metabolites and cytokines with a *r* > 0.99 and a *P*-value < 0.01 **(A)** Graph showing the average mitochondrial dimension in different mouse cohorts. (Tukey post ANOVA test: **P < 0.05 compared to all other groups of mice). **(B)** Graph showing the average surface density of mitochondrial envelop area in different mouse cohorts. (Tukey post ANOVA test: *P < 0.01 compared to all other groups of mice). **(C)** Graph showing the ROS levels in the liver of our different mouse cohorts. (Tukey post ANOVA test: *P < 0.05 compared to wild type and 0.4% treated *Gulo^−/−^* mice). **(D)** Correlation between liver ROS levels and serum ascorbate levels. **(E)** Correlation between serum 15S-HETE and the average mitochondrial dimension in mice. **(F)** Correlation between serum PAI-1 and the average mitochondrial dimension in mice. **(G)** Correlation between serum sE-selectin and the average surface density of mitochondrial envelop area in the liver of the different mouse cohorts. **(H)** Correlation between serum alpha-AAA and the average surface density of mitochondrial envelop area in the liver of the different mouse cohorts. The Pearson's correlation *r* values and *P*-values are indicated on each graph.

Since the mitochondrion is important in metabolism, we next examined the morphology of mitochondria in the liver of our mouse cohorts. Enlarged mitochondria in liver tissues have been reported in a mouse model of premature aging [24] and in human patients with liver disease [25]. Transmission electron microscopy revealed a significant increase in the dimension of hepatic mitochondria in ascorbate depleted *Gulo^−/−^* mice compared to wild type animals (Figure 7A). Supplementation of ascorbate in drinking water (0.01% or 0.4%) significantly decreased the size of the mitochondria to wild type dimensions. In contrast, the surface density of the mitochondrial envelop was significantly lower in all *Gulo^−/−^* mice (with 0%, 0.01% or 0.4% ascorbate) compared to wild type animals (Figure 7B; Tukey post-ANOVA test; *P* < 0.01). Finally, the number of mitochondria/hepatic surface area was not significantly different between cohorts of mice (supplementary Figure S4A and B; Tukey post-ANOVA test; *P* > 0.05). The levels of reactive oxygen species (ROS) were measured in the liver of mice. As indicated in Figure 7C, ROS level was increased in ascorbate depleted and 0.01% ascorbate treated *Gulo^−/−^* mice compared to wild type animals (Tukey post-ANOVA test; *P* < 0.05). ROS level was decreased in *Gulo^−/−^* mice treated with 0.4% ascorbate to wild type levels. Liver ROS levels significantly correlated inversely with serum ascorbate levels (Figure 7D).

We determined whether there were significant correlations between cytokines or metabolites and mitochondrial morphology in our mouse cohorts. Figure 7 (panels E-H) gives the molecules with the highest correlations (*r* > 0.99 and *P* < 0.01). The prostaglandin derivative 15S-HETE and the secreted protease PAI-1 were positively correlated with hepatic mitochondrial size (Figure 7E and F). Serum sE-selectin levels in return, correlated positively with the surface density mitochondrial envelop area (Figure 7G). In contrast, serum alpha-AAA levels correlated negatively with mitochondrial surface density envelop area (Figure 7H). Finally, hepatic mitochondrial size also significantly (*r* > 0.95; *P* < 0.05) correlated positively with histamine, spermidine, spermine, and 6-keto-PGF1a, and inversely with body weight, wet visceral fat weight, PC aa C42:4, and PC aa C42:6 (Table 3). The surface density mitochondrial envelop area significantly correlated with the ratio of C0/C4:1 and inversely with serum C16-OH levels and the Met-SO/Met ratio (Table 3).

**Table 3:** Pearson's correlation *r* values between median life span and organ wet weight

Average mitochondrial area (mm^2^)		
Cytokines or metabolites	*r* value	*P*-value
PAI-1	0.9934	< 0.01
Histamine	0.9696	< 0.05
Spermidine	0.9819	< 0.05
Spermine	0.9897	< 0.05
15S-HETE	0.9929	< 0.01
6-keto-PGF1a	0.9850	< 0.05
PC aa C42:4	−0.9534	< 0.05
PC aa C42:6	−0.9509	< 0.05
Body weight	−0.9746	< 0.05
Visceral wet weight	−0.9551	< 0.05
Surface density of mitochondrial envelop area (mm^−1^)		
Cytokines or metabolites	*r* value	*P*-value
sE-selectin	0.9987	< 0.01
C0/C4:1 ratio	0.9563	< 0.05
Met-SO/Met ratio	−0.9579	< 0.05
C16-HO	−0.9830	< 0.05
Alpha-AAA	−0.9973	< 0.01

### Effect of ascorbate on stress markers in the liver of *Gulo^−/−^* mice

**Figure 8:**
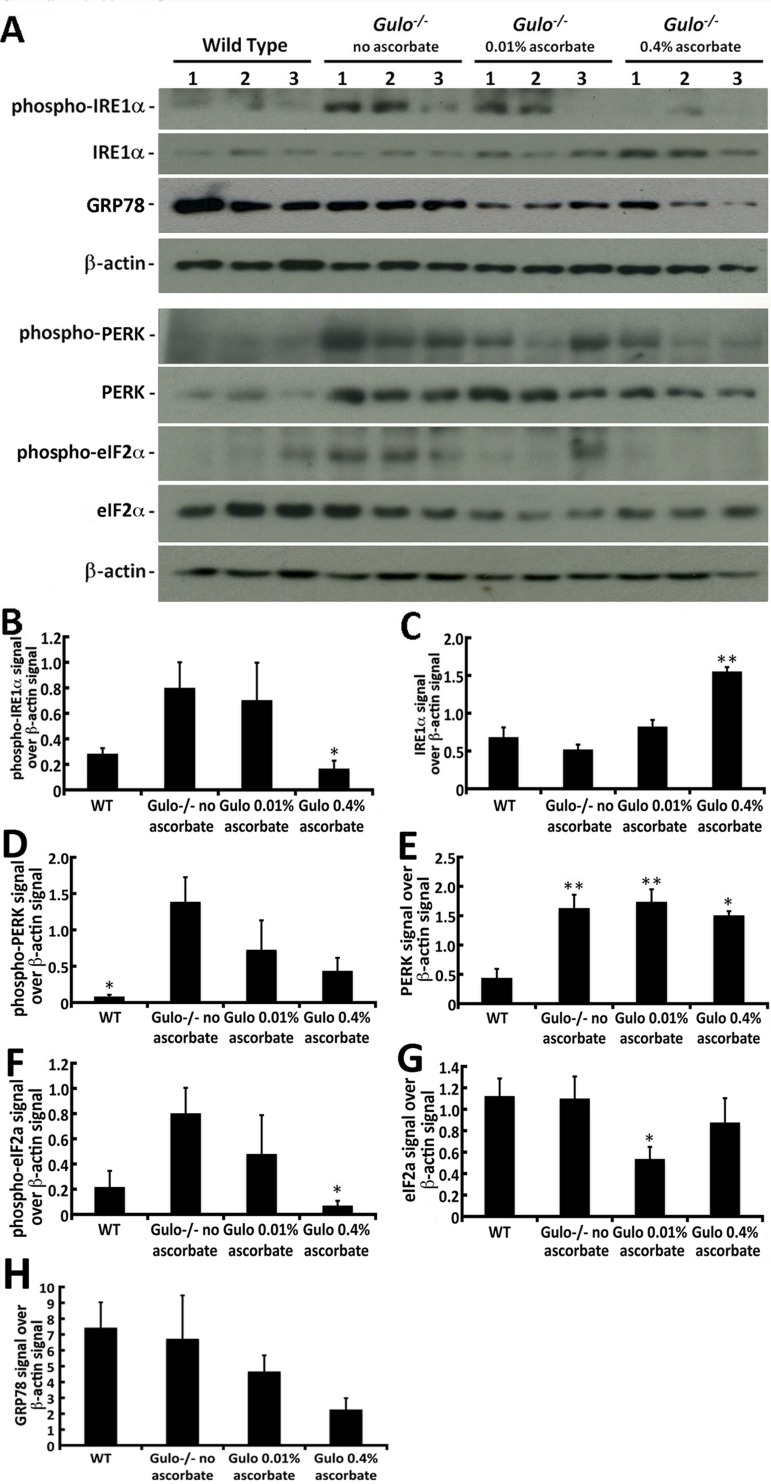
Impact of ascorbate on the levels of different ER stress markers in the liver of *Gulo^−/−^* mice **(A)** Example of western blots showing protein levels of phosphorylated and total IRE1α, GRP78, phosphorylated and total PERK, phosphorylated and total eIF2α. β-actin was used as loading controls. **(B)** Ratio of phosphorylated IRE1α signal over β-actin signal from the western blots. (ANOVA: *P* > 0.05; but student *t*-test: *P < 0.05 for 0.4% ascorbate treated *Gulo^−/−^* mice *vs*. ascorbate depleted *Gulo^−/−^* mice). **(C)** Ratio of total IRE1α signal over β-actin signal from the western blots. (Tukey post ANOVA test: **P < 0.01 compared to all other mouse groups). **(D)** Ratio of phosphorylated PERK signal over β-actin signal from the western blots. (Tukey post ANOVA test: *P < 0.05 compared to ascorbate depleted *Gulo^−/−^* mice). **(E)** Ratio of total PERK signal over β-actin signal from the western blots. (Tukey post ANOVA test: *P < 0.05 and **P < 0.01 compared to type mice). **(F)** Ratio of phosphorylated eIF2α signal over β-actin signal from the western blots. (ANOVA: *P* > 0.05; but student *t*-test: *P < 0.05 for 0.4% ascorbate treated *Gulo^−/−^* mice *vs*. ascorbate depleted *Gulo^−/−^* mice). **(G)** Ratio of total IRE1α signal over β-actin signal from the western blots. (ANOVA: *P* > 0.05 but student *t*-test: *P < 0.05 for 0.01% ascorbate treated *Gulo^−/−^* mice *vs*. wild type mice). (**H**) Ratio of total GRP78 signal over β-actin signal from the western blots. No significant difference was found between groups (ANOVA: *P* > 0.05; student *t*-test *P* > 0.05). Bars in all histograms represent SEM of three mice.

Since we could detect changes in serum inflammatory cytokines, lipids, and hepatic ROS in our different cohorts of mice, we analyzed the levels of several stress markers in the liver of these mice. Phosphorylation of IRE1α, PERK, and eIF2α were first analyzed by western blotting (Figure 8A). IRE1α acts as an endoplasmic reticulum (ER) stress sensor and is part of the unfolded protein stress response [26]. As indicated in Figure 8A and B, the levels of phosphorylated IRE1α protein were increased in the liver of *Gulo^−/−^* mice treated with 0.01% or depleted of ascorbate compared to wild type animals. Supplementation of 0.4% ascorbate in drinking water significantly reduced the levels of phosphorylated IRE1α proteins compared to ascorbate depleted *Gulo^−/−^* mice (Figure 8B; shown only by student *t*-test: *P* < 0.05). Total IRE1α, in return, was significantly increased in 0.4% ascorbate treated *Gulo^−/−^* mice compared to wild type, ascorbate depleted and 0.01% ascorbate treated *Gulo^−/−^* mice (Figure 8C; Tukey post-ANOVA test: *P* > 0.01). PERK is a transmembrane protein located in the ER and contains a kinase domain activated upon ER stress that phosphorylates eIF2α, thus inhibiting the translation of mRNAs [27]. ER stress also leads to PERK autophosphorylation. As indicated in Figure 8A and D, the levels of phosphorylated PERK was significantly increased in ascorbate depleted *Gulo^−/−^* mice compared to wild type animals (Tukey post-ANOVA or student *t*-test: *P* < 0.05). Total PERK levels were significantly increased in all *Gulo^−/−^* mice compared to wild type animals (Figure 8E; Tukey post-ANOVA or student *t*-test: *P* < 0.05). Phosphorylated eIF2α was significantly decreased in 0.4% ascorbate treated *Gulo^−/−^* mice compared to ascorbate depleted *Gulo^−/−^* mice (Figure 8A and F). Total eIF2α level was significantly decreased in 0.01% ascorbate treated *Gulo^−/−^* mice compared to wild type mice (based on student *t*- test: *P* < 0.05; Figure 8G).

GRP78, also referred as the immunoglobulin heavy chain-binding protein (BiP), is a member of the heat-shock protein-70 family and is involved in the folding and assembly of proteins in the ER [26]. There was a decrease in GRP78 in *Gulo^−/−^* mice treated with 0.4% ascorbate (Figure 8A), but overall there was no significant difference in the levels of this protein between groups of mice (Figure 8H; Tukey post-ANOVA or student *t*-test: *P* > 0.05).

**Figure 9:**
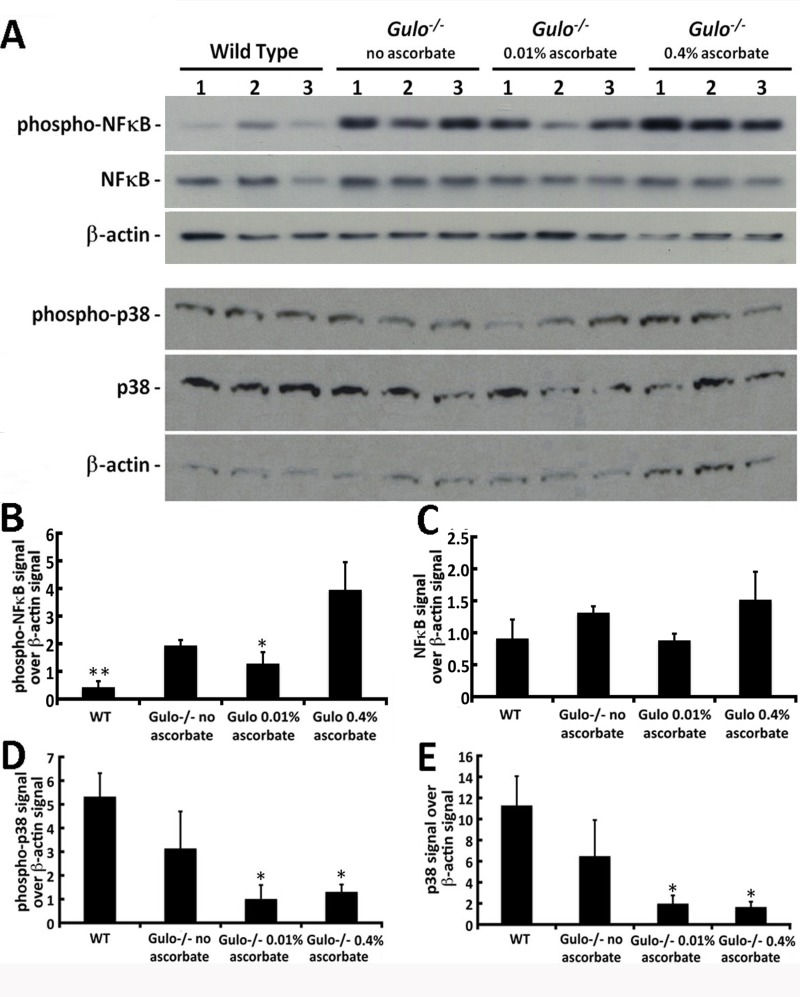
Impact of ascorbate on the levels of different stress markers in the liver of *Gulo^−/−^* mice **(A)** Example of western blots showing protein levels of phosphorylated and total NFκB, phosphorylated and total p38 MAP kinase. β-actin was used as loading controls. **(B)** Ratio of phosphorylated NFκB signal over β-actin signal from the western blots. (Tukey post ANOVA test: *P < 0.05 and **P < 0.01 compared to 0.4% ascorbate treated *Gulo^−/−^* mice). **(C)** Ratio of total NFκB signal over β-actin signal from the western blots. **(D)** Ratio of phosphorylated p38 MAP kinase signal over β-actin signal from the western blots. (Tukey post ANOVA test: *P < 0.05 compared to wild type mice). **(E)** Ratio of total p38 MAP kinase signal over β-actin signal from the western blots. (Tukey post ANOVA test: *P < 0.05 compared to wild type mice). Bars in all histograms represent SEM of three mice.

The NFκB transcription factor determines cell response to a wide variety of stresses, including inflammation, and was recently shown to be one of the most strongly age-associated markers [28]. As indicated in Figure 9A and B, phosphorylated NFκB levels were significantly different between groups of mice (Tukey post-ANOVA test: *P* > 0.01). Phosphorylation (and thus activation of NFκB) was increased in *Gulo^−/−^* mice treated with 0.4% ascorbate compared to wild type and *Gulo^−/−^* mice treated with 0.01% ascorbate. Total NFκB levels were similar in all groups of mice (Figure 9A and C).

We also examined the levels of total and phosphorylated p38 MAP kinase in the hepatic tissues of our mouse cohorts. As indicated in Figure 9D and E, the addition of ascorbate in drinking water decreased total and phosphorylated levels of p38 in the liver of *Gulo^−/−^* mice compared to wild type animals (Tukey post-post-ANOVA; *P* < 0.05). However, total and phosphorylated levels of p38 were not higher in the liver of ascorbate depleted *Gulo^−/−^* mice compared to wild type animals.

### Levels of phosphorylated IRE1α and eIF2α correlated with levels of ascorbate and median life span

We determined whether there were significant correlations between the stress markers analyzed in our mouse cohorts and the median life span or the serum ascorbate levels. Table 4 provides a summary of the correlations. Phosphorylated IRE1α and phosphorylated eIF2α were the only markers that correlated significantly (and inversely) with the median life span of mice in our different cohorts with a *P*-value of at least < 0.05. They also significantly correlated inversely with serum ascorbate levels (Table 4). Phosphorylated or total PERK, GRP78, NFκB, and p38 MAP kinase did not correlate significantly with median life span or ascorbate levels (Table 4). Thus, different phosphorylated markers of ER stress response correlated significantly with the median life span of *Gulo^−/−^* mice treated with different concentrations of ascorbate in drinking water.

**Table 4:** Pearson's correlation *r* values between liver stress markers and median life span or ascorbate

	Median life span	Ascorbate
Phospho-IRE1α/β; -actin ratio	−0.9842^†^	−0.9965*
Total IRE1α/β;-actin ratio	0.6563	0.6541
Phospho-PERK/β;-actin ratio	−0.9196	−0.8742
Total PERK/β;-actin ratio	−0.6011	−0.6111
Phospho-eIF2α/β;-actin ratio	−0.9840^†^	−0.9609^‡^
Total eIF2α/β;-actin ratio	0.2198	0.3051
GRP78/β;-actin ratio	−0.3468	−0.3273
Phospho-NFκB/β;-actin ratio	0.2141	0.2630
Total NFκB/β;-actin ratio	0.0943	0.1876
Phospho-p38/β;-actin ratio	0.1875	0.2321
Total p38/β;-actin ratio	0.1213	0.1596

* Pearson's correlation *r* values with a *P*-value < 0.01

† Pearson's correlation *r* values with a *P*-value < 0.02

‡ Pearson's correlation *r* values with a *P*-value < 0.05

### Correlation between stress markers and metabolites or cytokines

We next looked at the significantly altered metabolites or cytokines (Figures 2 and 4A) that correlated (with a *r* > 0.95 and a *P* < 0.05) with the different stress markers analyzed in the liver of our mouse cohorts. Significant correlations with the ER stress markers are shown in Table 5. Importantly, phosphorylated IRE1α and eIF2α correlated positively with the ratio of saturated to unsaturated phosphatidylcholines. Although the phosphorylation of PERK did not correlate significantly with median life span (only a tendency with a *r* = −0.9196), it positively correlated with the phosphorylation of eIF2α proteins. Total PERK inversely correlated with the surface density of mitochondrial envelop area (Table 5) and with several of the molecules that correlated with this mitochondrial parameter (like sE-selectin, C16-OH, alpha-AAA, and the Met-SO/Met ratio; compare Tables 3 and 5). The amount of the ER stress marker GRP78 correlated inversely with total IRE1α levels in the liver of our mouse cohorts. Finally, the levels of phosphorylated and total eIF2α correlated inversely with several lysophosphatidylcholine and phosphatidylcholine species in the serum of our mice (Table 5).

**Table 5:** Significant Pearson's correlation *r* values (with *P* < 0.05) between ER stress markers in the liver of our mouse cohorts and serum metabolites or cytokines

Metabolites, cytokines, stress markers	*r* value	*P*-value
Phosphorylated IRE1α/β-actin ratio		
Kynurenine	−0.9566	< 0.05
Tyr/Phe ratio	−0.9510	< 0.05
Saturated/unsaturated phosphatidylcholines	0.9672	< 0.05
Total IRE1α/β-actin ratio		
None significant		
Phosphorylated eIF2α/β-actin ratio		
Spermidine	0.9805	< 0.02
Lysophosphatidylcholine (acyl residue) C16:0	−0.9731	< 0.05
Lysophosphatidylcholine (acyl residue) C18:2	−0.9779	< 0.05
C-peptide 2	−0.9800	< 0.02
Saturated/unsaturated phosphatidylcholines	0.9942	< 0.01
Total IRE1α/β-actin ratio	0.9977	< 0.001
Phospho-IRE1α/β-actin ratio	0.9602	< 0.05
Total eIF2α/β-actin ratio		
Phosphatidylcholine; PC aa C36:0	−0.9683	< 0.05
Phosphatidylcholine; PC aa C38:5	−0.9633	< 0.05
Phosphatidylcholine; PC ae C38:3	−0.9573	< 0.05
Phosphatidylcholine; PC ae C40:0	−0.9961	< 0.01
Phosphatidylcholine; PC ae C30:1	−0.9902	< 0.01
Phosphatidylcholine; PC ae C42:0	−0.9799	< 0.05
Sphingomyelin SM C20:2	−0.9590	< 0.05
Phospho-PERK/β-actin ratio		
Glycine	0.9957	< 0.01
Phosphatidylcholine; PC aa C42:6	−0.9810	< 0.05
Phosphatidylcholine C36:0/C36:2 ratio	−0.9661	< 0.05
Phospho-eIF2α/β-actin ratio	0.9610	< 0.05
Total PERK/β-actin ratio		
Alpha-AAA	0.9956	< 0.01
C16-HO	0.9614	< 0.05
sE-selectin	−0.9969	< 0.01
Met-SO/Met ratio	0.9617	< 0.05
C0/C16-OH ratio	−0.9673	< 0.05
Surface density of mitochondrial envelop area	−0.9955	< 0.01
GRP78/β-actin ratio		
Phosphatidylcholine; PC aa C36:0	−0.9551	< 0.05
Leukotriene B4 (LTB4)	0.9644	< 0.05
Total IRE1α/β-actin ratio	−0.9954	< 0.01

Total and phosphorylated p38 correlated inversely with several phosphatidylcholine species including lipids with very long carbon chains in our different cohorts of mice (Table 6). Phosphorylated p38 also significantly correlated positively with total p38 in the liver of mice. Finally, phosphorylated NFκB did not significantly correlate with metabolites or cytokines.

**Table 6:** Significant Pearson's correlation *r* values (with *P* < 0.05) between p38 MAP kinase or NFκB stress markers in the liver of our mouse cohorts with serum metabolites or cytokines

Metabolites, cytokines, stress markers	*r* value	*P*-value
Phosphorylated p38/β-actin ratio		
Total p38/β-actin ratio	0.9958	< 0.01
Met-SO/Met ratio	−0.9612	< 0.05
Butenoylcarnitine (C4:1)	−0.9811	< 0.02
Phosphatidylcholine; PC ae C40:1	−0.9625	< 0.05
Phosphatidylcholine; PC ae C44:3	−0.9953	< 0.01
Phosphatidylcholine; PC ae C44:4	−0.9781	< 0.05
Total p38/β-actin ratio		
Phosphatidylcholine; PC ae C34:2	−0.9590	< 0.05
Phosphatidylcholine; PC ae C44:3	−0.9828	< 0.02
Phosphatidylcholine; PC ae C44:4	−0.9892	< 0.02
Leukotriene B4 (LTB4)	0.9571	< 0.05
Phospho-NFκB/β-actin ratio		
None significant		
Total NFκB/β-actin ratio		
None significant		

## DISCUSSION

The anti-oxidant and anti-inflammatory properties of ascorbate have been inversely correlated with several chronic diseases [29]. The impact of ascorbate on overall life span, however, has been inconsistent from one study to another [30-32]. Various confounding factors have been suggested to explain these discrepancies including sample size, dose duration, genetic variation, or disease status [33]. Despite the controversies surrounding the therapeutic potential of ascorbate in various chronic diseases, it is becoming clear that insufficient dietary vitamin C places individuals at greater risk for heart disease, cancer, or degenerative conditions [5, 8, 34, 35]. It has been reported that in the United States smokers and low-income persons suffer from varying degrees of ascorbate deficiency [5, 7]. Low dietary vitamin C may not necessarily lead to scurvy but suboptimal levels will impair the normal function of enzymes in several sub-cellular compartments like the nucleus, the endoplasmic reticulum, or the mitochondrion [36, 37]. Studies of the pathogenesis of human diseases related to vitamin C deficiency has benefited from the availability of mutant mice lacking the enzyme responsible for the last step of ascorbate synthesis [11]. In this study, we analyzed the impact of different concentrations of ascorbate on the serum levels of 203 metabolites by mass spectrometry and the serum concentrations of 42 cytokines involved in inflammation, cardiovascular diseases, or lipid metabolism in such mutant mice.

The median life span of *Gulo^−/−^* mice increased with the amount of ascorbate supplemented in the drinking water. Concomitantly, serum ascorbate levels in *Gulo^−/−^* mice increased with the amount of ascorbate in the diet. In fact, the median life span of the different cohorts of mice (including untreated wild type mice) correlated positively with the mean levels of ascorbate measured in the serum of each group of mice. Although we studied small cohorts of mice, it was clear that the maximum life span of *Gulo^−/−^* mice treated with 0.4% ascorbate was similar to untreated wild type animals. Higher concentrations of ascorbate were not tested in this study as we previously found that wild type mice treated with 0.4% of ascorbate did not exhibit an increase in their longevity compared to untreated wild type animals [18].

The absence of ascorbate in drinking water lead to a severe body weight loss in the *Gulo^−/−^* mouse as described previously [11, 17] with a concomitant decrease in visceral fat weight. This decrease in body mass is likely due to the observed decrease in food intake [15, 38]. Importantly, the decrease levels of hexoses and of most lysophosphatidylcholine and phosphatidylcholine molecules in the serum of ascorbate deficient *Gulo^−/−^* mice compared to age matched four-month old wild type animals reflected this phenotype. Supplementation of 0.01% or 0.4% ascorbate in drinking water increased the levels of these molecules to wild type levels or even above wild type concentrations, although ascorbate treated *Gulo^−/−^* mice were not significantly overweight compared to wild type animals (Figure 1C). The metabolic hormones C-peptide 2 and leptin were also significantly decreased in ascorbate deficient *Gulo^−/−^* mice (supplementary Figure S2). Supplementation of 0.01% or 0.4% ascorbate reversed such phenotype in *Gulo^−/−^* mice. However, the median life span and ascorbate levels did not significantly correlate with body weight, visceral fat weight, or with these two metabolic hormones. Apart from ascorbate, the only metabolite that highly correlated (*P* < 0.01) with the median life span of our cohorts under study was octadecadienoylcarnitine C18:2 (*r* = −0.9969). Octadecadienoylcarnitine C18:2 (or the linoleyl carnitine) is a long-chain acyl fatty acid derivative ester of carnitine. Long-chain acyl fatty acid derivatives are known to accumulate in the cytosol and serum of patients suffering from mitochondrial carnitine palmitoyltransferase II deficiency, the most common inherited disorder of lipid metabolism in adults [39]. Octadecadienoylcarnitine is also known to inhibit the mitochondrial complex IV resulting in an increase in reactive oxygen species [40]. Accordingly, *Gulo^−/−^* mice with low levels of serum ascorbate exhibited increased ROS in their liver compared to age-matched wild type animals.

Resistin was the only cytokine significantly correlating (*P* < 0.01) with the median life span of mice in our cohorts (*r* = −0.9912). Interestingly, resistin also correlated with serum ascorbate levels in our different cohorts of mice (Figure 5D). Resistin is a cytokine produced by fat and immune cells that can modulate the obesity pro-inflammatory environment [41]. Resistin has been described as a cytokine that links obesity to diabetes in mice [42]. Importantly, it has been reported that ascorbate supplementation in humans significantly reduces resistin levels, presumably through its antioxidant activity, and this is independent of changes in inflammatory or other metabolic variables [43]. The results with our *Gulo^−/−^* cohorts recapitulate the data from this report. Finally, low resistin levels have been associated with low prevalence of metabolic syndrome and healthier centenarians [44]. Thus, a low serum resistin level in ascorbate treated *Gulo^−/−^* mice would be in agreement with a longer life span. However, despite these interesting observations in our mouse cohorts, the difference in serum C18:2 and resistin levels between groups of mice was not statistically significant based on Kruskal-Wallis analyses.

We also examined the ratios of different metabolites and these showed significant differences between our groups of mice and identified potential metabolic pathways significantly correlated with median life span. Eight specific ratios were significantly different between our groups of mice (based on ANOVA analyses, Figure 6). Four metabolite ratios correlated significantly with the median life span (Table 1). The Tyr/Phe ratio correlated positively with median life span. Hydroxylation of phenylalanine to tyrosine is a pathway affected by ascorbate concentrations *in vivo* [20]. Accordingly, we found a significant correlation between Tyr/Phe ratio and serum ascorbate levels. The Arg/Lys ratio also correlated positively with median life span. Arginine is required for nitric oxide synthesis, a molecule with cardiovascular protective properties. Since lysine competes with arginine for the same intracellular transporter [21], this ratio provides an indirect assessment of arginine bioavailability for nitric oxide synthesis and cardioprotection. In this context, the low Arg/Lys ratio in ascorbate depleted *Gulo^−/−^* mice is in agreement with the aortic wall damage observed in these mice when treated with low levels of ascorbate [11].

Interestingly, the ratio of saturated/unsaturated phosphatidylcholines and more specifically the ratio of PC aa C36:0/PC aa C36:2 phosphatidylcholines correlated significantly with median life span. The life span of *Gulo^−/−^* mice treated with different concentrations of ascorbate did not correlate with body weight or visceral wet weight. This indicates that in *Gulo^−/−^* mice the ratio of saturated/unsaturated lipids is a more relevant determinant of life span than total serum lipids.

We observed that the dimensions of hepatic mitochondria increased in ascorbate depleted *Gulo^−/−^* mice. Supplementation of 0.01% ascorbate was enough to reverse mitochondrial dimensions to wild type size (Figure 7). Hepatic mitochondrial size correlated significantly with serum levels of 15S-HETE and PAI-1. Interestingly, it has been reported that PAI-1 levels can regulate mitochondrial mass in cancer cells [45]. The positive correlation between PAI-1 and mitochondrial dimension in liver of ascorbate depleted *Gulo^−/−^* mice is consistent with such findings. The impact of 15S-HETE on mitochondrial size is unknown. However, 15S-HETE possesses anti-proliferative properties. Increased serum levels of 15S-HETE may reflect a response to the abnormal mitochondrial morphological dimension and increased ROS observed in ascorbate depleted *Gulo^−/−^* mice. Mitochondrial dimension also correlated positively with histamine, spermine, and spermidine. In contrast, hepatic mitochondrial size correlated inversely with specific long chain phosphatidylcholines (PC aa C42:4 and PC aa C42:6), body weight and visceral fat weight. Interestingly, it has recently been reported that excess visceral adiposity is significantly associated with mitochondrial size and dysfunction [46]. Spermine and spermidine are known to modulate hepatic mitochondrial metabolism [47]. Histamine, in return, has been shown to affect the size of mitochondria of gastric cells in starved guinea pigs [48].

The surface density of mitochondrial envelop area significantly correlated positively with serum sE-selectin levels and the serum ratio of C0/C4:1. It correlated inversely with serum levels of alpha-AAA, C16-OH, and serum Met-SO/Met ratio. Carnitine (C0) and the acylcarnitine C4:1 and C16-OH species may likely affect mitochondrial envelop as they are important metabolites for the transport of fatty acids in the mitochondria during lipid metabolism. The impact alpha-AAA and sE-selectin on surface density of mitochondrial envelop area is unknown. Alpha-AAA (alpha-aminoadipic acid) is increased in all *Gulo^−/−^* mice. It is an intermediate in the degradation of lysine. Thus, an increase of serum alpha-AAA may represent an impaired turnover of decarboxylation 2-oxoadipate to glutaryl-CoA, which is the last step in the lysine degradation pathway [49]. Aging, diabetes, sepsis, and renal failure are known to catalyze the oxidation of lysyl residues to alpha-AAA in human skin collagen and potentially other tissues. Proteolytic breakdown of these tissues can lead to the release of free alpha-AAA [50]. Dysfunctional endothelial cells leads to elevated levels of alpha-AAA, which is also thought to be a sign of lysyl residues breakdown in proteins through oxidative stress and reactive oxygen species [51]. We also observed a significant inverse correlation between serum alpha-AAA and serum sE-selectin (Figure 5). E-selectin is expressed on cytokine-activated endothelial cells and contributes to the adhesion of leukocytes to the endothelium and activation of the immune cells. After cell activation, E-selectin is eliminated from the cytoplasmic membrane by shedding into the circulation as secreted E-selectin (or sE-selectin). It is possible that in ascorbate depleted *Gulo^−/−^* mice, cell damage reflected by the increased serum alpha-AAA and abnormal surface density of mitochondrial envelop area leads to sE-selectin response required for the elimination of irreversibly damaged cells. Injection of these molecules in *Gulo^−/−^* mice will be required to determine the cause from effect.

Addition of ascorbate in the diet reversed the decreased life span of *Gulo^−/−^* mice. However, even though *Gulo^−/−^* mice treated with 0.4% ascorbate had a life span similar to wild type animals, their overall metabolic profile was still different from the wild type profile. The PCA analysis clearly indicated that *Gulo^−/−^* mice treated with either 0.01% or 0.4% ascorbate clustered together and did not overlap with the wild type animals (Figure 3). Thus, ascorbate did not re-establish a complete wild type metabolic profile. In contrast, the cytokine profile of *Gulo^−/−^* mice treated with 0.4% ascorbate overlapped with that of the wild type animals (Figure 4B).

Although we could detect changes in the levels or the activation of different stress markers in the liver of ascorbate depleted *Gulo^−/−^* mice, the phosphorylated ER stress associated markers IRE1α and eIF2α were the only proteins that highly correlated (and inversely) to the mean life span of *Gulo^−/−^* mice treated with different concentrations of ascorbate. Phosphorylated IRE1α and eIF2α proteins also inversely correlated significantly with serum ascorbate levels in our different mouse cohorts, suggesting that the ER stress can be alleviated by ascorbate. Accordingly, it has been reported that ascorbate protects against metal-induced ER stress in male gonads of mice [52]. Phosphorylated IRE1α also significantly correlated inversely with the Tyr/Phe and saturated/unsaturated phosphatidylcholine ratios in our mouse cohorts. Unbalanced fatty acid ratio is known to affect membrane lipid composition [53] and may thus affect the ER membrane, trans-membrane transporters, or membrane associated enzymatic activities in *Gulo^−/−^* mice. Phosphorylation and thus activation of PERK during ER stress leads to eIF2α phosphorylation and inhibition of mRNA transcription. As expected, we found a significant positive correlation between activation of PERK and phosphorylation of eIF2α. However, unlike the phosphorylation of eIF2α, we did not detect a significant correlation (only a tendency) between phospho-PERK levels and the median life span of our mouse cohorts. Importantly, phosphorylation of eIF2α can occur independently of PERK activity in cells [54] or tissues upon inflammation or oxidative stress [55], a possibility that will need further investigation in our aging mouse cohorts. Interestingly, total PERK levels correlated inversely with the surface density of mitochondrial envelop area in our mouse cohort. It has been reported that PERK is an essential component of the mitochondria-associated ER membranes that establish a physical and functional connection between the ER and the mitochondria. PERK serves also as a structural tether at the ER-mitochondria interface regulating inter-organellar cross-talk in ROS-induced cellular response [56]. It is thus possible that *Gulo^−/−^* mice increased total PERK protein levels in response to the alterations observed in the surface density of mitochondrial envelop area in our mouse cohorts. Finally, we found that other stress markers like the p38 MAP kinase and the transcription factor NFκB did not correlate with the life span of ascorbate treated *Gulo^−/−^* mice.

To conclude, our study demonstrates the impact of suboptimal levels of ascorbate on longevity and the metabolic profile of a mouse model, that similar to humans, lacks the enzyme required for the synthesis of ascorbate. Vitamin C supplementation alleviated the abnormal levels of several lipid species (Figure 2) and cardiovascular risk factors (Figure 4 and Table 1). Finally, serum ascorbate levels and longevity correlated significantly with the ER stress response as seen by the activation of IRE1α and inhibition of eIF2α through phosphorylation reactions. As such, this study is the first work reporting a significant correlation between ER stress response and longevity in *Gulo^−/−^* mice treated with different concentrations of ascorbate.

## MATERIALS AND METHODS

### Animals and maintenance

*Gulo^−/−^* mice were obtained from the Mutant Mouse Regional Resource Centers (University of California Davis, CA) and were housed at the Centre de Recherche de l'Hôtel-Dieu de Québec animal facility. This study was carried out in strict accordance with the recommendations in the Guide for the Care and Use of Laboratory Animals of the Canadian Council on Animal Care in science. The protocol was approved by the Committee on the Ethics and Protection of Animal of Laval University (Permit Number: 2014029). Mice were housed in cages (containing a top filter) at 22 ± 2°C with 40%–50% humidity and a 12-h light–dark cycle (light cycle: 06:00–18:00 hours). All mice were fed *ad libitum* with Teklad Global (Madison, WI) 18% protein rodent diet, 5% fat, and 0% vitamin C (135 IU/kg of vitamin E and 30 IU/g of vitamin A). Euthanasia was performed by treating mice with 3% isoflurane (general anesthesia) followed by cervical dislocation. Animals were checked every day for any external mass, infection, bleeding, gasping, and overall decrease or change in activity or behavior. Mice that lost 20% of total body weight, became immobile, or moribund were sacrificed for histological examination of their organs as described previously [57]. One cohort of *Gulo^−/−^* mice was maintained on standard diet and supplemented with 0.4% of L-ascorbate (Sigma-Aldrich, Oakville, ON) (w/v) in drinking water from weaning until the age of four months. A second cohort of *Gulo^−/−^* mice was maintained on standard diet and supplemented with 0.01% of L-ascorbate (w/v) in drinking water from weaning until the age of four months. A third cohort of *Gulo^−/−^* mice was maintained on standard diet and supplemented with 0.01% of L-ascorbate (w/v) in drinking water from weaning until the age of three months. Ascorbate was then removed from drinking water for four weeks. Wild type control C57BL/6 mice were maintained in the same room with no ascorbate supplementation in drinking water and were used as our normal reference. The analyses were performed on four month-old animals as more than 85% of *Gulo^−/−^* mice treated with 0.01% ascorbate were healthy at that age.

### Serum collection for analysis

Blood was harvested at 10:00 am by cardiac puncture and exsanguination under anesthesia at the age of four months. Blood was allowed to clot for one hour at 4°C and spun on a bench top centrifuge at maximum speed for 15 min. Serum was collected and frozen at −80°C until execution of analyses. Ascorbic acid in serum was measured with the ferric reducing ascorbate assay kit from BioVision Research Products (Mountain View, CA, USA).

### Metabolite measurements

Metabolite measurements were performed by the BIOCRATES Life Sciences metabolomic services (BIOCRATES Life Sciences AG, Innsbruck, Austria). Briefly, Biocrates’ commercially available kit plates were used for the quantification of amino acids, acylcarnitines, sphingomyelins, phosphatidylcholines, hexoses, and biogenic amines. The fully automated assay was based on PITC (phenylisothiocyanate)-derivatization in the presence of internal standards followed by FIA-MS/MS (acylcarnitines, lipids, and hexose) and LC/MS (amino acids, biogenic amines) using an AB SCIEX 4000 QTrap® mass spectrometer (AB SCIEX, Darmstadt, Germany) with electrospray ionization. The experimental metabolomics measurement technique has been previously described [53]. Eicosanoids and other oxidized polyunsaturated fatty acids were extracted from samples with aqueous acetonitrile that contained deuterated internal standards. The metabolites were determined by HPLC-tandem mass spectrometry (LC-MS/MS) with Multiple Reaction Monitoring (MRM) in negative mode using a SCIEX API 4000 QTrap mass spectrometer with electrospray ionization. The LC-MS/MS method used for the analytical determination of eicosanoids has been published [58]. Accuracy of the measurements (determined with the accuracy of the calibrators) was in the normal range of the method (deviations from target ≤ 20 %) for all analytes. In total, 203 different metabolites were measured. The metabolomics data set contains 21 amino acids, 19 biogenic amines, one hexose (H1), free carnitine (C0), 40 acylcarnitines (C*x:y*), hydroxylacylcarnitines (C(OH)*x:y*), and dicarboxylacylcarnitines (C*x:y*-DC), 15 sphingomyelins (SM*x:y*) and *N*-hydroxylacyloyl-sphingosylphosphocholine (SM (OH)*x:y*), 77 phosphatidylcholines (PC, aa = diacyl, ae = acyl-alkyl), 14 *lyso*-phosphatidylcholines, and 17 eicosanoid acids and prostaglandins. Lipid side chain composition is abbreviated as C*x:y*, where *x* denotes the number of carbons in the side chain and *y* the number of double bonds. For example, “PC ae C30:1” denotes an acyl-alkyl phosphatidylcholine with 30 carbons in the two fatty acid side chains and a single double bond in one of them [53]. Full biochemical names are provided in Supplementary Table S1 and the raw data in the Supplementary Tables S2. Note that the precise position of the double bonds and the distribution of the carbon atoms in different fatty acid side chains cannot be determined with this technology.

### Cytokine measurements

The following cytokines were assessed in the serum (diluted 1:4) using the multiplex kit from Bio-Rad Laboratories Canada Ltd. (catalogue number MD000000EL; Mississauga, ON, Canada): IL-15, IL18, LIF, M-CSF, MIG, MIP-2, PDGF-BB, VEGF, and FGF basic. The following cytokines were assessed in the serum (diluted 1:4) using the multiplex kit from Bio-Rad (catalogue number MD60009RDPD): IL-1α, IL-1β, IL-2, IL3, IL-4, IL-5, IL-6, IL-10, IL-12(p40), IL-12(p70), IL-13, IL-17, KC, MCP-1, MIP-1α, MIP-1β, TNF-α, Rantes, Eotaxin, G-CSF, GM-CSF, and IFN-γ. The metabolic hormones C-peptide 2, GIP, Leptin, and Resistin were assessed in the serum using the Milliplex Mouse Metabolic Magnetic Bead Panel (catalogue number MMHMAG-44K) from EMD Millipore Corp. (Billerica, MA, USA). The following cardiovascular risk factors were assessed in the serum (diluted 1:20) using the Milliplex Mouse CVD Panel 1 kit from EMD Millipore Corp. (catalogue number MCVD1MAG-77K): soluble E-selectin, P-selectin, ICAM-1, Pecam-1, proMMP-9, thrombomodulin, and total PAI-1. Full biological names are provided in Supplementary Table S3. All measurements were performed on a Bio-Plex® 200 system (with Bio-Plex Manager™ software version 6.0) from Bio-Rad Laboratories Canada Ltd. (Mississauga, ON, Canada).

### Morphological and ultrastructural studies of liver tissues

For liver morphology studies, liver perfusion was performed as described previously [59]. Following fixation, liver samples for transmission electron microscopy were embedded in Spurrs resin, sectioned, and examined using a Philips CM10 transmission electron microscope. Liver morphology was assessed for mitochondrial density, mitochondrial size, surface density of mitochondrial envelop using ImageJ software. Three to five mice per genotype were analyzed by electron microscopy techniques. Fifteen micrographs per animal of hepatocytes cytoplasm were taken at 9,900 × magnification. Mitochondria were manually counted and the number of mitochondria per cytoplasmic unit area was calculated. Mitochondria area was measured by tracing around five random mitochondria in each micrograph. The surface density of mitochondrial envelope (Sv) was estimated using the formula Sv=2*I/Lt where I represents the number of intersections of mitochondria envelops with parallel lines (1μm apart) and Lt the total length of lines as described [60]. Scanning electron microscopy was performed as previously described [61]. Ten images (magnification x 20,000) were taken for each animal for analysis of fenestration diameter and endothelial porosity.

### Reactive oxygen species (ROS) measurements in liver tissue

Liver lysates in RIPA buffer (50 mM Tris-HCl (pH 7.5), 150 mM NaCl, 1% NP-40, 0.1% SDS, 0.5% sodium deoxycholate, 30 mM NaF, 60 mM glycerophosphate, 20 mM sodium pyrophosphate, 1 mM sodium orthovanadate, 1mM phenylmethyl-sulfonylfluoride and complete protease inhibitor cocktail (Roche Applied Science, Indianapolis, IN)) were incubated with 10 μg/mL of the dye 2′-7′ dichlorofluorescein diacetate (Sigma-Aldrich Canada Ltd, Oakville, ON) for 1h at 37°C. This dye is highly fluorescent upon oxidation. As control, RIPA buffer was also incubated with dichlorofluorescein diacetate and 100 μL (500 μg of liver proteins) of the samples were put into 96-well plates. Fluorescence was measured with a Fluoroskan Ascent fluorescence spectrophotometer (Thermo Electron Inc., Milford, MA). The excitation and emission wavelengths used were 485 and 527 nm, respectively. Background fluorescence was extracted from the dichlorofluorescein value for each sample and the final result was expressed as units of fluorescence per gram of proteins.

### Western blotting

All steps were performed on ice or at 4°C. Tissue protein extraction was carried out in lysis buffer containing 20 mM MOPS (pH 7.0), 2 mM EGTA (pH 8.0), 5 M EDTA (pH 8.0), 30 mM NaF, 60 mM glycerophosphate (pH 7.2), 20 mM sodium pyrophosphate, 1 mM sodium orthovanadate, 3 mM benzamidine, 5 μM pepstatin A, 10 μM leupeptin, 0.5% Triton X-100, 1 mM DTT, 1mM phenylmethyl-sulfonylfluoride and complete protease inhibitor cocktail (Roche Applied Science, Indianapolis, IN). Proteins were then resolved on a 4-12% Criterion XT Bis-Tris gradient gel (Bio-Rad, Mississauga, ON, Canada) and then transferred onto 0.2 μm PVDF membrane (EDM Millipore Corp., Temecula, CA). After incubating 1 h with blocking solution (PBS-T containing 5% nonfat milk), the membrane was probed overnight at 4°C with a primary antibody. After washing with PBS-T, species-specific horseradish peroxidase-conjugated secondary antibody was added for 2 h at room temperature (GE Healthcare Limited, Piscataway, NJ). Signals were generated with Western Lightning Chemiluminescence reagent plus kit (GE Healthcare Limited, Piscataway, NJ). When indicated, immunoblots were probed with the following antibodies: rabbit polyclonal antibodies raised against the eukaryotic translation initiation factor 2-α kinase 3 (anti-PERK (H300): sc-13073), eukaryotic translation factor 2- α (anti-eIF2 α (FL-315): sc-11386), phosphorylated eIF2 α (anti-p-eIF2 α (Ser 52): sc101670) from Santa Cruz Biotechnology (Santa Cruz, CA); a rabbit polyclonal antibody against phospho-inositol-requiring kinase 1α (anti-phospho-IRE1α, NB100-2323) from Novus Biologicals (Burlington, ON, Canada); rabbit monoclonal antibodies against inositol-requiring kinase 1α (anti-IRE1 #3294), phospho-NFκB (anti-phospho-NFκB p65 (Ser536) (93H1)), NFκB (anti-NFκB p65 (D14E12)), and phosphorylated PERK (anti-phospho-PERK (Thr980) (16F8)) from Cell Signaling Technology (Beverly, MA); rabbit polyclonal antibodies against phospho-p38 (anti-phospho-p38 MAP Kinase (Thr180/Tyr182)) and total p38 (anti-p38 MAP Kinase) from Cell Signaling Technology (Beverly, MA); a rabbit polyclonal antibody against glucose-related protein 78 (anti-GRP78) from Proteintech^TM^ (Chicago, IL); a mouse monoclonal antibody against actin (A5441) from Sigma-Aldrich (Oakville, ON).

### Statistical analyses

Identification of significantly different metabolites was achieved by using the Kruskal-Wallis nonparametric test. Principal component analysis (PCA) and presentation of the results using heatmaps were performed using R versions 2.14. To take into account multiple testing, we considered significant events using a *P* < 0.01. Heatmaps were generated using Euclidean distance and complete agglomerative methods. One-way ANOVA followed by Tukey's HSD (honest significant difference) Test for post-ANOVA pair-wise comparisons were performed for the morphological studies and for the western blot analyses. Differences were considered significant at a *P*-value < 0.05. These tests were calculated using the http://faculty.vassar.edu/lowry/ank3.html website. Pearson's correlation *r* values were calculated with Excel. For our colony of four different groups of mice, the *r* value must be greater than 0.95 to reach significance with a *P*-value < 0.05.

## SUPPLEMENTAL DATA FIGURES AND TABLES










