# Supplementary material for: Vitamin C modulates the metabolic and cytokine profiles, alleviates hepatic endoplasmic reticulum stress, and increases the life span of Gulo−/− mice
Source: Aging (Albany NY). 2016 Feb 20;8(3):458–83. doi: 10.18632/aging.100902 (PMC4833140; doi:10.18632/aging.100902)
Supplement: Supplementary file 1 [file aging-08-458-s001.docx]

**SUPPLEMENTARY DATA**


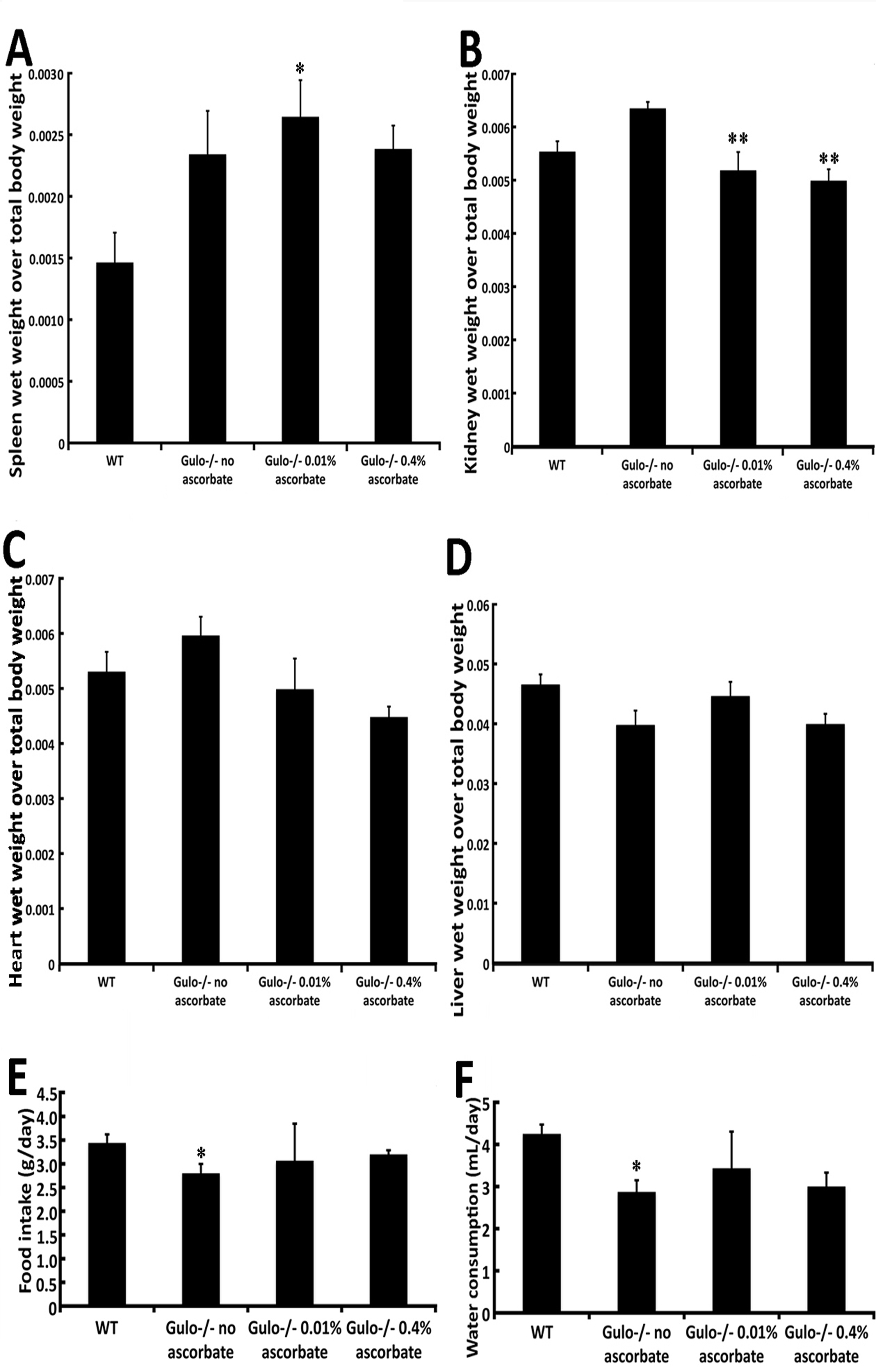


**Figure S1.** **Impact of ascorbate on the weight of different organs in *Gulo^-/-^* mice treated with different amounts of ascorbate. (A)** Histogram representing the ratio of spleen wet weight over total body weight. (Tukey post ANOVA test **P* < 0.05 compared to wild type mice). **(B)** Histogram representing the ratio of kidney wet weight over total body weight. (Tukey post ANOVA test ***P* < 0.01 compared to ascorbate depleted *Gulo^-/-^* mice). **(C)** Histogram representing the ratio of heart wet weight over total body weight. **(D)** Histogram representing the ratio of liver wet weight over total body weight. **(E)** Histogram representing food intake. (ANOVA: *P* > 0.05 but student *t*-test: **P* < 0.05 compared to wild type mice). **(F)** Histogram representing water consumption. Bars in all histograms represent SEM. (ANOVA: *P* > 0.05 but student *t*-test: **P* < 0.05 compared to wild type mice). One cohort of *Gulo^-/-^* mice was treated with 0.01% of ascorbate (w/v) in drinking water from weaning to four months of age. A second cohort of *Gulo^-/-^* mice was treated with 0.4% of ascorbate from weaning to four months of age. In a third cohort of *Gulo^-/-^* mice, ascorbate was omitted from the drinking water at the age of three months for four weeks. Wild type mice were not treated with vitamin C. N=6 males for each cohort.

www.impactaging.com 1 AGING, February 2016, Vol. 8 No.2


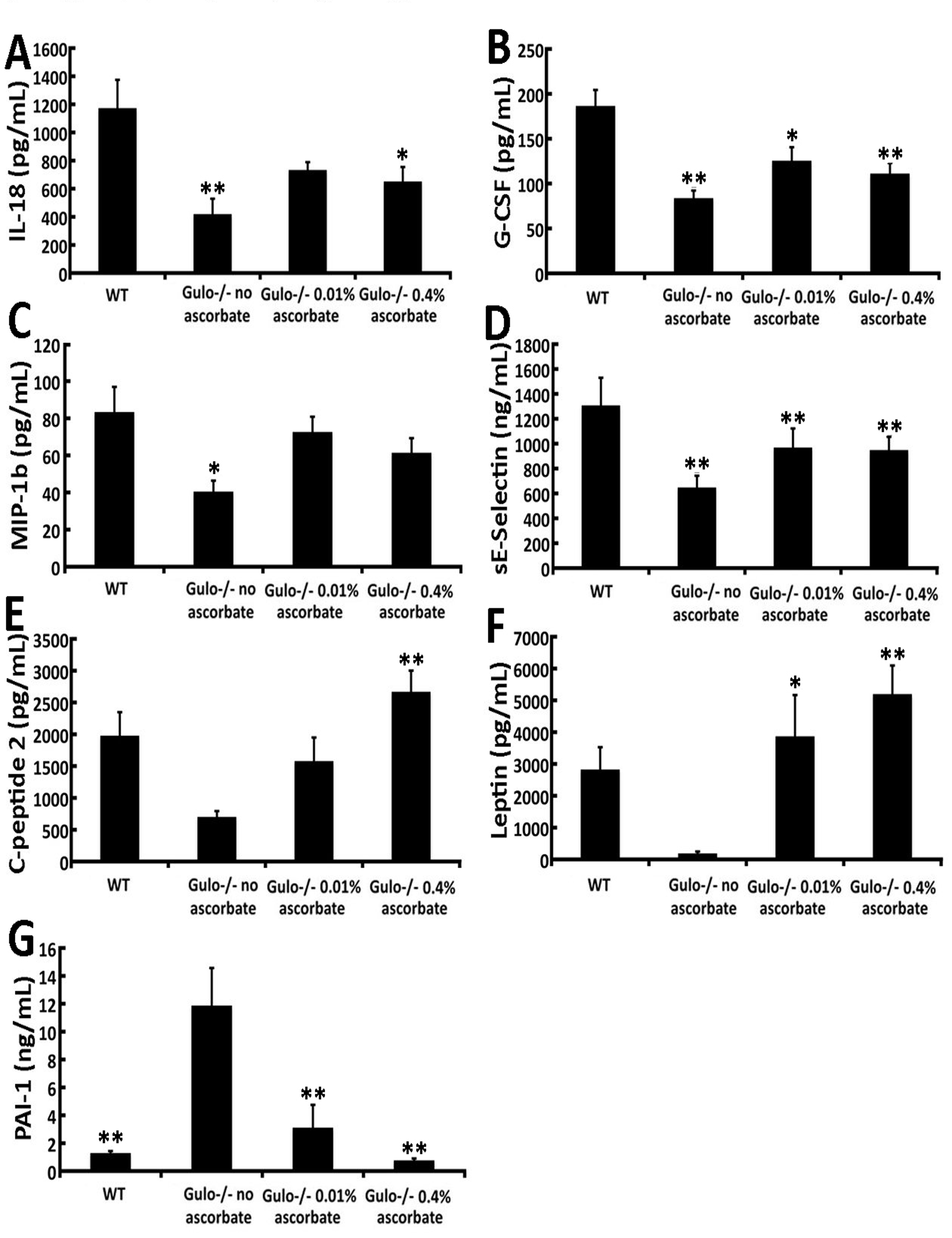


**Figure S2. Serum level of seven serum cytokines significantly altered in *Gulo^-/-^* mice treated with different amount of ascorbate. (A)** IL-18 (Tukey post ANOVA test: **P* < 0.05 and ***P* < 0.01 compared to wild type mice). **(B)** G-CSF (Tukey post ANOVA test: **P* < 0.05 and ***P* < 0.01 compared to wild type mice). **(C)** MIP-1b (Tukey post ANOVA test: **P* < 0.05 compared to wild type mice). **(D)** sE-Selectin (Tukey post ANOVA test: ***P* < 0.01 compared to wild type mice). **(E)** C-peptide 2 (Tukey post ANOVA test: ***P* < 0.01 compared to ascorbate depleted *Gulo^-/-^* mice). **(F)** Leptin (Tukey post ANOVA test: **P* < 0.05 and ***P* < 0.01 compared to ascorbate depleted *Gulo^-/-^* mice). **(G)** PAI-1 (Tukey post ANOVA test: ***P* < 0.01 compared to ascorbate depleted *Gulo^-/-^* mice). One cohort of *Gulo^-/-^* mice was treated with 0.01% of ascorbate (w/v) in drinking water from weaning to four months of age. A second cohort of *Gulo^-/-^* mice was treated with 0.4% of ascorbate from weaning to four months of age. In a third cohort of *Gulo^-/-^* mice, ascorbate was omitted from the drinking water at the age of three months for four weeks. Wild type mice were not treated with vitamin C. N=8 males for each cohort.

www.impactaging.com 2 AGING, February 2016, Vol. 8 No.2

**Figure S3.** **Impact of ascorbate on sinusoidal endothelial fenestration in *Gulo^-/-^* mice treated with different amount of ascorbate. (A)** Frequency of fenestrae/surface hepatocyte area in the different cohorts of mice. **(B)** Average fenestration diameter in the different cohorts of mice. **(C)** Examples of scanning electron microscopy showing lumenal surface of the liver sinusoidal endothelium with fenestrations clustered into sieve plates in wild type and *Gulo^-/-^* mice treated with different concentrations of ascorbate.

www.impactaging.com 3 AGING, February 2016, Vol. 8 No.2


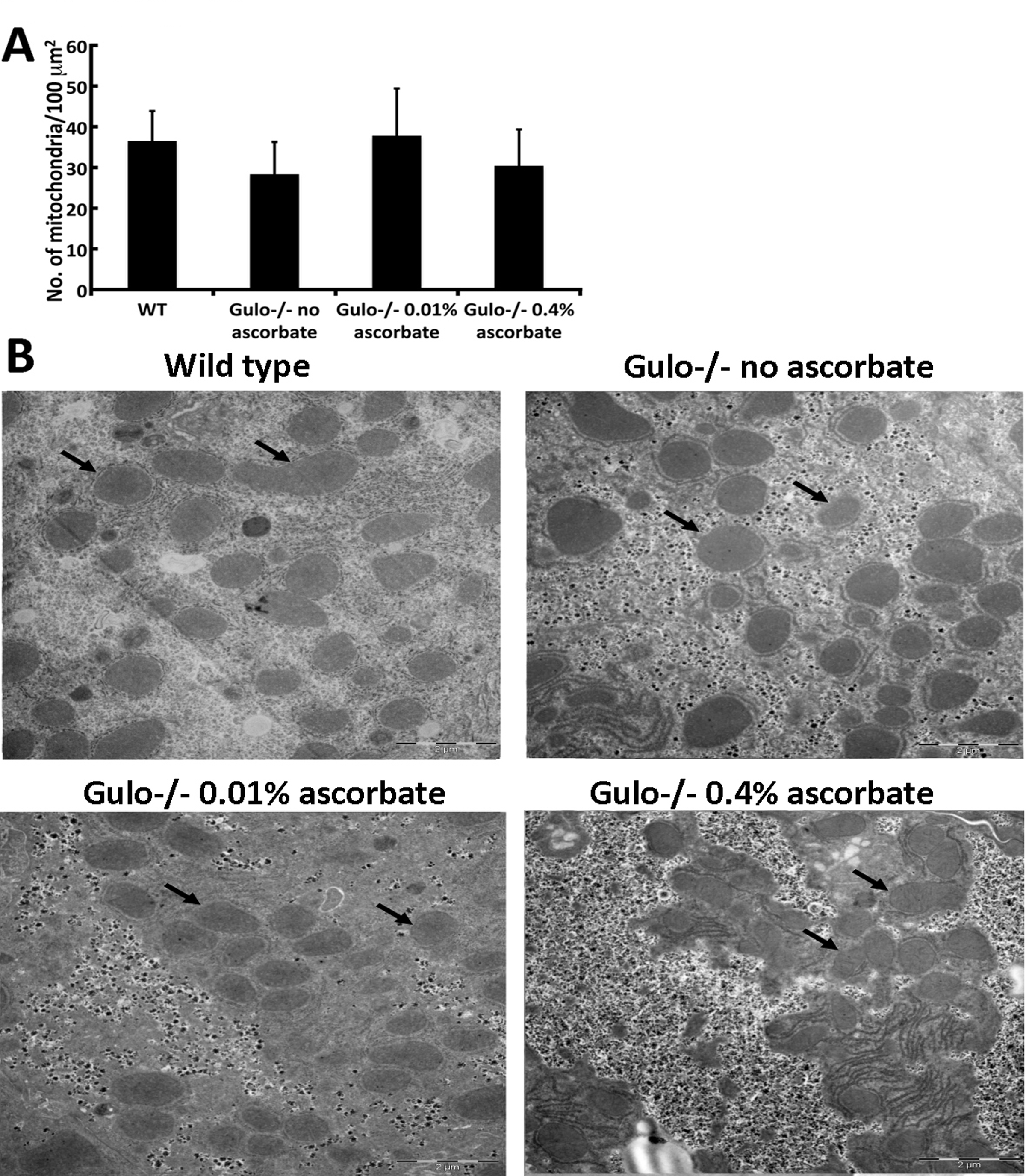


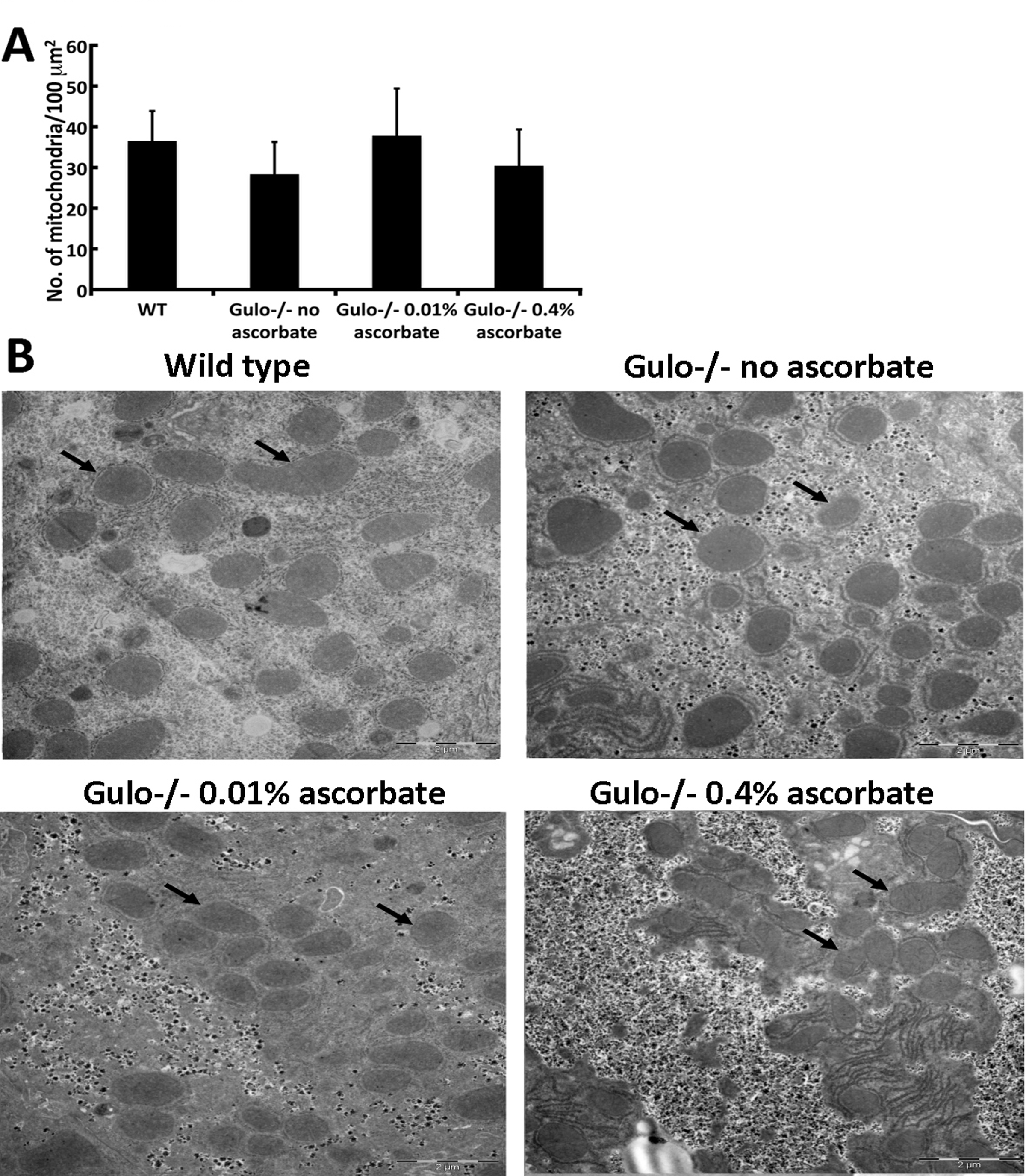


**Figure S4.** **Impact of ascorbate on the number of mitochondria/hepatocyte surface area in *Gulo^-/-^* mice treated with different amount of ascorbate. (A)** Graph showing the frequency of mitochondria/hepatic surface area. **(B)** Examples of transmission electron microscopy showing mitochondrial size in wild type and *Gulo^-/-^* mice treated with different concentrations of ascorbate. Arrows point to mitochondria.

www.impactaging.com 4 AGING, February 2016, Vol. 8 No.2
